# A hippocampo-cerebellar centred network for the learning and execution of sequence-based navigation

**DOI:** 10.1038/s41598-017-18004-7

**Published:** 2017-12-19

**Authors:** Benedicte M. Babayan, Aurélie Watilliaux, Guillaume Viejo, Anne-Lise Paradis, Benoît Girard, Laure Rondi-Reig

**Affiliations:** 1 Sorbonne Universités, UPMC Univ Paris 06, INSERM, CNRS, Neurosciences Paris Seine - Institut de Biologie Paris Seine (NPS - IBPS), Cerebellum Navigation and Memory team (CeZaMe), 75005 Paris, France; 2 Sorbonne Universités, Université Pierre et Marie Curie (UPMC), CNRS UMR 7222, Institut des Systèmes Intelligents et de Robotique (ISIR), F-75005 Paris, France

**Keywords:** Learning algorithms, Hippocampus, Cerebellum, Neural circuits, Navigation

## Abstract

How do we translate self-motion into goal-directed actions? Here we investigate the cognitive architecture underlying self-motion processing during exploration and goal-directed behaviour. The task, performed in an environment with limited and ambiguous external landmarks, constrained mice to use self-motion based information for sequence-based navigation. The post-behavioural analysis combined brain network characterization based on c-Fos imaging and graph theory analysis as well as computational modelling of the learning process. The study revealed a widespread network centred around the cerebral cortex and basal ganglia during the exploration phase, while a network dominated by hippocampal and cerebellar activity appeared to sustain sequence-based navigation. The learning process could be modelled by an algorithm combining memory of past actions and model-free reinforcement learning, which parameters pointed toward a central role of hippocampal and cerebellar structures for learning to translate self-motion into a sequence of goal-directed actions.

## Introduction

Goal-directed behaviour allows generating action sequences to achieve a desired outcome^1–4^. Here, we wanted to focus on the neural substrate sustaining the acquisition of such a sequence in a navigation context: from the exploration of the environment to the successful performance of a sequence of goal-directed actions.

Possible principles for learning how to perform an ordered sequence of actions at successive discrete choice points are suggested by the firing properties of hippocampal pyramidal cells. For example, the forward replay of place cells that occurs at the subject’s current location^5^ and simulates the sequence of places to be visited en-route to the goal location has been proposed to reflect the use of model-based learning processes^6,7^. When occurring in reverse order, during quiet wakefulness or sleep, replay was proposed to contribute to evaluate events in a manner that engages model-free reinforcement learning rules (by back-propagating information about the reward)^8,9^. Common to both model-based and model-free classes of reinforcement learning is the idea that we can represent the world as a set of states at which actions can be taken. Yet, whereas model-based learning allows inferring possible future outcomes before acting through explicit representations of dependencies in the world representation, model-free learning does not rely on such forward-looking inference and is stimulus-driven^10^. Alternatively, hippocampal pyramidal cells’ activity may also directly code for time and distance (from departure) thus reflecting path integration^11^. It has been described with “time cells”^12,13^ or place cells paced by self-motion^14^. We previously proposed that the process of acquiring sequence-based navigation may rely on the hippocampus in both mice^15,16^ (reviewed in^17^) and humans^18,19^. Knowing that CA3 inputs have a greater influence than entorhinal cortex inputs on CA1 neural activity in sequence-based navigation compared to place-based navigation^14^, it is likely that the hippocampus performs a specific memory-driven operation when navigation requires a temporal organization of events and/or relies on self-motion information^20,21^. However, these studies did not address how this specific memory-driven functioning of the hippocampus arises with learning or to what extent it relies on interactions with other brain structures.

Interacting with the hippocampus, other players have been proposed in the network for sequence-based navigation and are thought to code different processes than the spatial location and temporal ordering contributed by the hippocampus. Prefrontal cortical neurons, for instance, have been implicated in coding task rules^22,23^ and goal locations^24^. The ventral striatum has been proposed to integrate hippocampal and prefrontal information to construct outcome predictions that invigorate motivated behaviours and support their selection^25–27^. We previously found an implication of the dorsomedial striatum in mice and caudate nucleus and right cerebellum Crus I in humans in sequence-based navigation^16,18,28^. The implication of the basal ganglia on one hand in sequence-based navigation, is compatible with human studies of route recognition and memory^29–31^, and orients the possible learning rules underlying acquisition of a sequence-based memory toward reinforcement learning^32,33^. The cerebellum on the other hand is classically proposed to perform supervised learning^34^. However, it has also been recently proposed to participate in the step of prediction of state-action-state transitions, used in model-based reinforcement learning^35^, or to encode temporal-difference prediction error^36^, a reinforcement learning teaching signal^37^.

**Figure 1:**
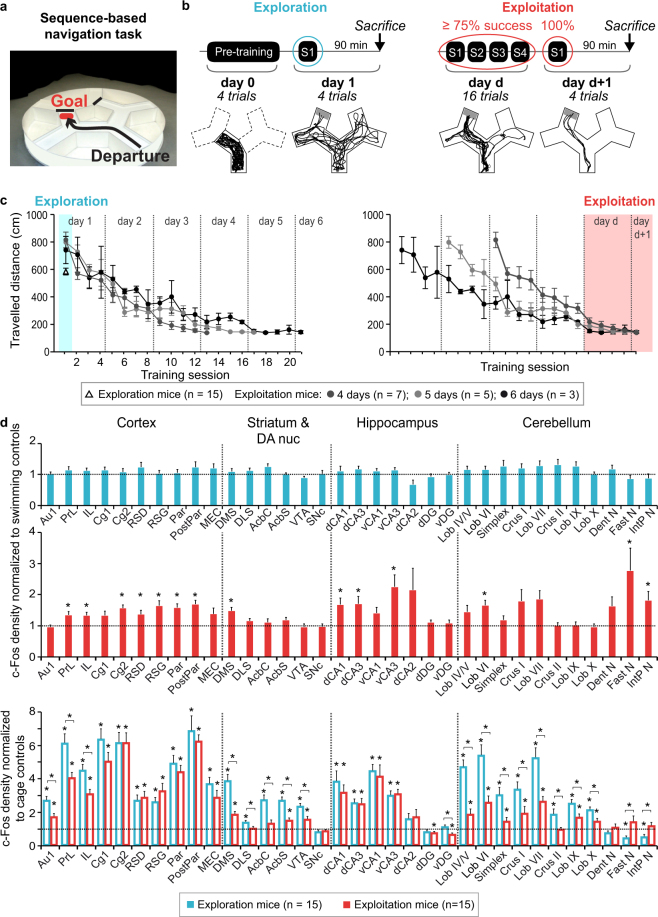
Behaviour and activation patterns across exploration and exploitation of sequence-based memory. (**a**) Mice learned a two-turn path in the sequence-based navigation task. (**b**) Left panel: Exploration mice did a pre-training session and the following day one training session during which they discovered the task. Below are shown the four trajectories of one exploration mouse for pre-training and session 1. Right panel: exploitation mice did 4 daily sessions until performing over 75% successful trials in one training day and 100% successful trials in one session the following day. Below are shown an exploitation mouse’s trajectories (13/16 correct trials, 4/4 correct trials the following day). (**c**) Exploration (Δ; left) and exploitation mice (●) travelled distance per session, aligned to the first session (left) or to the respective exploitation mice’s last session (right). Exploitation mice reached the exploitation criterion within 4, 5 or 6 training days and had comparable performances on their respective last two training days (right panel) (p > 0.2 for Kruskal-Wallis comparisons). Data represents mean ± s.e.m. (**d**) Top and middle: c-Fos positive cell densities for exploration and exploitation mice normalized to their respective swimming controls. Bottom: To compare c-Fos positive cell densities for exploration and exploitation mice, they are normalized to cage controls. Swimming and cage controls averages are indicated by the dotted horizontal lines. Exploration mice did not show any difference with their swimming controls, yet most structures had increased c-Fos positive cell densities compared to cage control mice. Exploitation mice showed increased activity in several areas, mostly cortical and hippocampal. The deep cerebellar fastigial and interpositus nuclei had a unique pattern of decreased activity in exploration mice compared to cage controls and increased c-Fos positive cell density in exploitation mice. *indicate significant differences (q < 0.05, FDR corrected, Mann Whitney comparisons). Data represents mean ± s.e.m. Abbreviations: cortex: primary auditory (Au1), prelimbic (PrL), infralimbic (IL), cingulate 1 and 2 (Cg1, Cg2), dysgranular and granular retrosplenial (RSD, RSG), parietal and posterior parietal (Par, PostPar), medial entorhinal (MEC); striatum and dopaminergic nuclei (DA nuc): dorsomedial striatum (DMS), dorsolateral striatum (DLS), nucleus accumbens core (AcbC) and shell (AcbS), ventral tegmental area (VTA), substantia nigra pars compacta (SNc); hippocampus: dorsal CA1 (dCA1), dorsal CA3 (dCA3), ventral CA1 (vCA1), ventral CA3 (vCA3), dorsal CA2 (dCA2), dorsal and ventral dentate gyrus (dDG, vDG); cerebellum: lobules IV/V (Lob IV/V), VI (Lob VI), VII (Lob VII), IX (Lob IX), X (Lob X), Simplex (Spx), dentate (Dent N), fastigial (Fast N) and interpositus nuclei (IntP N).

Despite this extensive literature, the learning processes underlying the acquisition of sequence-based navigation and the structures interacting with the hippocampus to sustain this acquisition remain uncertain. Here we aim at describing the brain networks involved in the ‘exploration phase’, when self-motion signals are used to gather information about the environment and in the ‘exploitation phase,’ when actions at choice points are organized in sequence and directed toward a goal. We thus trained mice to learn a path composed of successive turns leading to an invisible goal in an environment with limited environmental landmarks (Fig. 1a). Imaging-based approaches can detect coordinated activity across distributed and spatially remote brain regions, and therefore have been useful in defining functional networks^38–40^. We thus identified the brain networks associated with exploring this environment and performing sequence-based navigation using c-Fos imaging combined with graph theory analysis. Then, by comparing three computational learning models to the experimental learning dynamics, we identified brain structures that could sustain learning of the sequence-based behaviour by correlating their c-Fos activity with individual learning parameters.

## Results

### A navigation task to study acquisition of sequence-based memory

Mice had to swim to a platform hidden beneath the water surface in a maze composed of three Y-intersections surrounded by a black round curtain regularly rotated (Fig. 1a). In the exploration stage, mice were naive with respect to the goal of the task. This exploration stage not only involved the spatial exploration of the maze, but also early learning of the task’s rule. To reach the exploitation stage, mice had to reproducibly swim along the optimal path of two turns leading to the platform. Thirty mice were trained up to six days (16 trials per day) until they were able to achieve over 75% correct trials during one training day and 100% on a recall session of four trials the following day (Fig. 1b right). This exploitation criterion was chosen to ensure a similar level of task mastery by all exploitation mice. Fifteen mice reached this stage (exploitation group, Fig. 1c). The exploitation phase was the earliest time when mice were able to efficiently use the learned sequence, without reaching an overtrained plateau yet (Fig. 1c, right). All mice in the exploitation group had comparable performances on their last two training days (p > 0.2 for all Kruskal-Wallis comparisons), whether they reached the criterion on day 4 (n = 7), day 5 (n = 5) or day 6 (n = 3) (Fig. 1c, right).

### Widespread activations during exploration, more specific activations during exploitation

We identified the structures activated in the exploration and exploitation phases of sequence-based navigation by measuring the expression of the activity-regulated gene c-Fos in 34 regions (Supplementary Fig. 1).

To study the exploration phase, we measured c-Fos expression in a group of fifteen mice after one training session of four trials (Fig. 1b and c, left). To avoid this activity being mainly accounted for by stress and task novelty^41^, those mice completed on the previous day a pre-training session consisting in swimming four trials in a two-alley corridor without a platform (Fig. 1b left). To ensure the pre-training session without platform would not dramatically alter the exploration behaviour of exploration mice in the full maze compared to that of exploitation mice, we compared speed and travelled distance between the training sessions following a pre-training session (exploration group, ‘Session 1’ in Fig. 1b) and those following a classical training session (exploitation group, ‘Session2’ in Fig. 1b). For speed, the analysis of variance with group (n = 2) as independent factor and trials (n = 4) as repeated measurement factor revealed neither main effect of the group (F_1,28_ = 1.7, p > 0.2) nor interaction between group and trials (p > 0.11). By contrast, there was a significant effect of trials (F_3,84_ = 2,076, p < 0.001), which corresponds to the speed decreasing across trials in both groups. For travelled distance, the (2 × 4) repeated-measure ANOVA revealed neither main effect of the group (F_1,28_ = 2.06, p > 0.16) nor interaction between group and trials (F_3,84_ = 0.32, p > 0.8). With no significant difference between groups and a similar pattern of speed decrease across trials, these results overall suggest that the pre-training did not impact the way exploration mice adapted their behaviour at the beginning of the training in the whole maze, compared to the exploitation mice.

The observation that exploitation mice, which did not have a pre-training session, travelled a longer distance than exploration mice to reach the platform on the first training session (unpaired t-test, t_28_ = −4.99, p < 0.01; Fig. 1c left) further suggests that the pre-training session efficiently minimized the contribution of swimming novelty and stress in exploration mice.

To study the exploitation phase, we measured c-Fos expression in the fifteen mice that reached the exploitation criterion (Fig. 1b and c, right, Supplementary Fig. 2 for staining examples).

To control for non-specific c-Fos expression^41^, we normalized the raw c-Fos positive cell counts of each brain structure with two control measures: the mean cell count obtained in paired swimming control mice (specific control groups for exploration and exploitation groups) and in cage control mice (see Materials and Methods).

We first tested the c-Fos activity of the exploration and exploitation mice normalized to their respective control group (Fig. 1d top), and then tested and compared their activity normalized with the cage control group (Fig. 1d bottom). We found the exploration group did not have c-Fos activity significantly different from their paired swimming control group (Fig. 1d top, Supplementary Table 1). By comparing the exploration group and their paired swimming controls, we aimed to test whether the slight differences of context (larger environment, presence of a platform and choice points) would be sufficient to highlight specific activity related to exploring and encoding a complex environment compared to a more restricted one. Our results rather suggest that, in the time range of one session, the main effect on c-Fos density is related to the common explorative functions used by both groups. Yet, when normalized to cage control mice, exploration mice and their paired swimming controls (Supplementary Fig. 3, Supplementary Table 3) appeared to have significantly increased activities in 27 regions including cortical and striatal areas, in the ventral tegmental area (VTA), in the dorsal and ventral hippocampal CA1 and CA3 and cerebellar cortices compared to cage controls. Two cerebellar nuclei, the fastigial and interpositus nuclei had decreased density compared to cage control mice (Kruskal-Wallis, *q* < 0.05, FDR corrected, followed by Mann-Whitney tests, *q* < 0.05, FDR corrected; Fig. 1d bottom, Supplementary Table 2).

In the exploitation group, significant increases in c-Fos positive cell densities compared to their paired swimming control group were found in fourteen regions. Twelve of them were part of the 27 regions found activated in exploration mice compared to cage controls, namely the prelimbic, infralimbic, cingulate 2, dysgranular and granular retrosplenial, parietal and posterior parietal cortices, the dorsomedial striatum, the dCA1, dCA3, vCA3, and the cerebellar lobule VI (Kruskal-Wallis, *q* < 0.05, FDR corrected, followed by Mann-Whitney tests, *q* < 0.05, FDR corrected; Fig. 1d middle, Supplementary Table 1).

In addition, the two regions, which had decreased c-Fos density in exploration mice compared to cage controls, the fastigial and the interpositus nuclei, had increased density in exploitation mice, compared to their paired swimming controls and cage controls (Kruskal-Wallis, *q* < 0.05, FDR corrected, followed by Mann-Whitney tests, *q* < 0.05, FDR corrected; Fig. 1d middle and bottom, Supplementary Table 1, Supplementary Table 2 and Supplementary Fig. 2).

When directly comparing the exploitation and exploration groups (Fig. 1d bottom) normalized to the cage controls, eleven regions had similar c-Fos densities in both groups (cingulate, retrosplenial, parietal, entorhinal cortices and hippocampal CA1 and CA3 regions) and seventeen regions were found significantly more activated in the exploration group (prefrontal and striatal regions as well as cerebellar cortices). Reciprocally, only the fastigial and interpositus nuclei had significantly higher densities in exploitation mice compared to exploration mice (Kruskal-Wallis, *q* < 0.05, FDR corrected, followed by Mann-Whitney tests, *q* < 0.05, FDR corrected; Fig. 1d bottom, Supplementary Table 2 and Supplementary Fig. 2).

A decrease of activity in primary auditory cortex was observed between exploration and exploitation phases (Mann-Whitney test, q < 0.05, FDR corrected; Fig. 1d bottom, Supplementary Table 1). The noise being irrelevant to the task, the auditory activity likely becomes meaningless when mice learn the task (hence the decrease). Importantly, no difference was observed within stages (exploration or exploitation) between the test mice and their associated swimming control groups (Mann-Whitney test, q > 0.9, FDR corrected; Supplementary Table 1).

We examined possible differences between left and right hemispheres. However, no laterality effect was found in either exploration or exploitation mice (Sign test left versus right, *q* > 0.2, FDR corrected; Supplementary Table 4).

In summary, among the many structures activated during the exploration phase compared to cage control mice, only a subset was still activated when the mice performed sequence-based navigation. Amongst these, hippocampal regions were activated in both phases and maintained similar levels of activation. Two structures were specifically activated at the exploitation stage only, the fastigial and interpositus nuclei, two cerebellar outputs.

### From a predominant cortico-striatal network during exploration to a network driven by hippocampo-cerebellar interaction during exploitation

**Figure 2:**
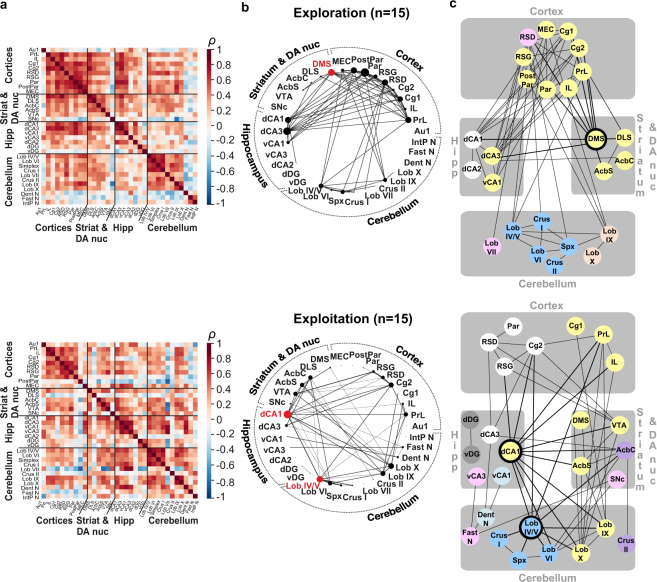
Functional network of the acquisition of a sequence-based memory. (**a**) Inter-regional correlation matrices for exploration (top) and exploitation (bottom) mice, each normalized to their respective controls. Axes correspond to brain structures. Colours reflect correlation strength (scale, right). (**b**) Network graphs generated by considering only the strongest correlations (Spearman’s ρ ≥ 0.64, p ≤ 0.01), with the thickness of the connections proportional to correlation strength and node size proportional to degree. Network hub structures are highlighted in red. The exploration mice’s network appears to be centred around cortical correlations with striatal, hippocampal and cerebellar structures, with the dorso-medial striatum as a network hub. The exploitation network is dominated by hippocampo-cerebellar correlations, with two network hubs, the hippocampal dorsal CA1 and cerebellar lobules IV/V. (**c**) Markov clustering algorithm was applied to organize brain structures into discrete color-coded modules based on their common inter-connections. Network hubs are highlighted in black. Consistent with the network graph analysis, the clustering in the exploration network revealed a major cortico-striatal cluster with the hub (in yellow), also containing hippocampal regions, and alongside three regionally confined clusters (a hippocampal cluster and two cerebellar clusters). In the exploitation network, the clustering revealed an interregional cluster with cortical, striatal, hippocampal and cerebellar structures (in yellow), alongside a cortico-hippocampal cluster (in white) and several other inter-regional clusters. The two exploitation network hubs (highlighted in black) belong to two different clusters and are at the interface of several other clusters, illustrating their central position in the network. Abbreviations: cortex: primary auditory (Au1), prelimbic (PrL), infralimbic (IL), cingulate 1 and 2 (Cg1, Cg2), dysgranular and granular retrosplenial (RSD, RSG), parietal and posterior parietal (Par, PostPar), medial entorhinal (MEC); striatum and dopaminergic nuclei (DA nuc): dorsomedial striatum (DMS), dorsolateral striatum (DLS), nucleus accumbens core (AcbC) and shell (AcbS), ventral tegmental area (VTA), substantia nigra pars compacta (SNc); hippocampus: dorsal CA1 (dCA1), dorsal CA3 (dCA3), ventral CA1 (vCA1), ventral CA3 (vCA3), dorsal CA2 (dCA2), dorsal and ventral dentate gyrus (dDG, vDG); cerebellum: lobules IV/V (Lob IV/V), VI (Lob VI), VII (Lob VII), IX (Lob IX), X (Lob X), Simplex (Spx), dentate (Dent N), fastigial (Fast N) and interpositus nuclei (IntP N).

It has been proposed that, during learning, a set of structures can be mobilized as a functional network corresponding to coherent neural activity^38–40^. To further identify such clusters among the set of brain regions analysed, we next examined the correlation of activity between all pairs of structures, separately for the exploration and exploitation groups, using c-Fos positive cell density normalized to their respective swimming controls (Fig. 2). Correlation matrices were generated by computing Spearman’s correlations between all pairs of structures across individual mice (Fig. 2a). To generate the network graphs, only the strongest positive correlations between structures were considered (applied threshold: Spearman’s ρ ≥ 0.64, corresponding to a two-tailed significance level of p ≤ 0.01) (Fig. 2b).

Exploration and exploitation groups respectively revealed 71 and 55 significant functional correlations, which distributions greatly differed. During exploration, cortical regions were involved in most of the significant correlations (72%) (Fig. 2b, top). By contrast, the exploitation group mainly displayed correlations involving the dorsal hippocampus and cerebellar cortices. Indeed, the dCA1 of the hippocampus, lobules IV/V, VI, IX and X were each correlated to respectively 11, 9, 6, 7 and 8 structures, out of 33 possible, and contributed 58% (32 out of 55) of the significant correlations in the exploitation group (Fig. 2b, bottom).

Importantly, the inter-area correlations described above at p ≤ 0.01 were maintained in networks generated using both less and more conservative thresholds (respectively low confidence network: p ≤ 0.05; ρ ≥ 0.51 and high confidence network: p ≤ 0.002; ρ ≥ 0.73; Supplementary Fig. 4).

To identify the most influential structures, we then searched for possible *hubs* using a conjunction of two criteria: the number of connections (degree, illustrated by node size in Fig. 2b) and the number of short communication paths the structure participates in (betweenness)^38^. To ensure the robustness of the procedure, hubs had to rank over the 80^th^ percentile for both criteria together in the main network (p ≤ 0.01), and in the low- and high-confidence networks^38^. We thus identified one hub region in the exploration group, the dorso-medial striatum and two hubs in the exploitation network: dCA1 and cerebellar lobules IV/V (highlighted in red in Fig. 2b and with black circle in Fig. 2c, Supplementary Fig. 5a and b). To further test the possible dependence of hub identification on one specific individual, we repeated the exact same hub identification procedure across 15 leave-one-out networks for each group, i.e. removing one of the 15 mice each time (Supplementary Fig. 5c). For exploration mice, 7 of the 15 leave-one-out networks did not reveal any hubs, and possible hubs appeared with lower frequency (maximum 4/15). For exploitation mice in comparison, there were only 4 leave-one-out networks with no hub, while the dorsal CA1 and lobule IV/V were hubs in 6 and 8 leave-one-out networks respectively.

To further characterize the structure of the networks, we applied a Markov clustering algorithm^42^, which organizes the overall network by grouping structures into clusters to maximize connections within clusters with respect to connections between clusters. This analysis was also consistent with a major network reorganization occurring over learning (Fig. 2c).

Five clusters were identified in the exploration group (Fig. 2c, top): a cortico-striato-hippocampal (yellow), a cortico-cerebellar (pink), a hippocampal (white) and two cerebellar clusters (salmon and blue).

The exploitation network was divided into seven clusters (Fig. 2c, bottom) with one major inter-regional component (yellow) involving structures belonging to four major anatomically segregated brain regions: prefrontal cortex areas (infralimbic, prelimbic, cingulate 1), medial areas of the striatum (dorsomedial striatum and accumbens shell), the VTA, the dCA1 and lobules IX and X of the cerebellum. Another cluster involved the dCA3 with retrosplenial cortices (dysgranular and granular), the parietal cortex and area 2 of the cingulate cortex. Two clusters involved the ventral hippocampus (vCA3 and vCA1) and respectively the fastigial and dentate nuclei of the cerebellum.

Both networks had a common cerebellar cluster composed of lobules IV/V, VI, Simplex and Crus I. This cluster had few (5) correlations with other clusters in the exploration group but exhibited an increased number of significant correlations (10) in the exploitation mice with hippocampal, cortical and striatal regions, through lobules IV/V, a hub region, and the lobule VI.

Only the exploitation network contained clusters (3/7) gathering cerebellar and hippocampal regions.

To ensure the robustness of the network analyses with respect to the c-Fos normalization, we repeated graph network, hub and cluster analysis on c-Fos densities normalized on cage controls (Supplementary Fig. 6a and c) and on the difference of raw c-Fos density between experimental mice and their paired swimming controls (Supplementary Fig. 6b and d). For exploration mice, the cortico-striatal centred feature was maintained in both cases, with hubs being either striatal or cortical (Supplementary Fig. 6a and b). For exploitation mice, we again observed a reduction of cortico-striatal connections in favour of hippocampo-cerebellar connections, with in both cases a cerebellar hub (Supplementary Fig. 6c and d).

For the sake of completeness, we also analysed the networks of swimming control mice, either normalized to themselves or to cage controls (Supplementary Fig. 7). The exploration swimming control mice’s networks appeared similar to exploration mice’s network, but with more significant correlations at p ≤ 0.01 (Supplementary Fig. 7a and b). Exploitation swimming control networks by contrast had sparse significant correlations, mostly within cortical and cerebellar regions (Supplementary Fig. 7c and d). Interestingly, exploitation swimming control mice revealed the same cerebellar cluster common to exploration and exploitation groups, containing notably the lobules IV/V, VI, Simplex and Crus I.

Overall, hippocampo-cerebellar correlated activity across mice was a feature only observed when the animals achieved fluency with the task in the exploitation phase.

In summary, the networks observed in exploration and exploitation groups showed major differences. The functional network of mice in the exploration phase, a phase in which mice discover various task features such as the structure of the maze, the rule, was dominated by correlated activity centred around cortico-striatal regions whereas the main feature of exploitation mice was a network centred on the dorsal hippocampus and cerebellar cortex (in particular lobules IV/V). These two structures were involved in numerous correlations with each other and the rest of the network when mice were exploiting sequence memory to perform optimal navigation.

### Hippocampus and cerebellum activity correlates with exploration/exploitation trade-off of a model-free reinforcement learning with memory of past actions

To determine which brain structures could sustain the acquisition of the sequence behaviour, we correlated c-Fos activity with individual learning parameters. Those learning parameters were obtained by testing the ability of three classes of computational learning algorithms classically used to model navigation tasks to capture the mice’s behaviour. We compared 1) a learning model using path integration, which relies on identifying the position of the platform relative to the starting point by integrating displacements; 2) model-based reinforcement learning, which relies on building a graph representation of the successive choice points in the maze to plan the shortest path to the platform at the beginning of each trial; 3) model-free reinforcement learning, which associates the most rewarding action (the most likely to lead to the platform) to each choice point. Since the choice points were perceptually similar, we tested two versions of the model-free reinforcement learning model: a classical model-free reinforcement algorithm, which does not distinguish choice points, and a model-free reinforcement learning algorithm with a memory of past actions to distinguish the choice points from one another based on self-motion information. All models included three parameters: η, the learning rate; β, the exploitation-exploration trade-off; and γ, the discount factor for reinforcement learning models or σ, the cumulative error on position estimation for path integration.

For each mouse and for each model, we identified the set of so-called “optimal” parameters reproducing the mouse’s decisions with the highest likelihood (Supplementary Table 5). The comparison across models revealed that the learning dynamics of all mice were best fit by model-free reinforcement learning, either with or without a memory of past actions (Supplementary Table 5).

**Figure 3:**
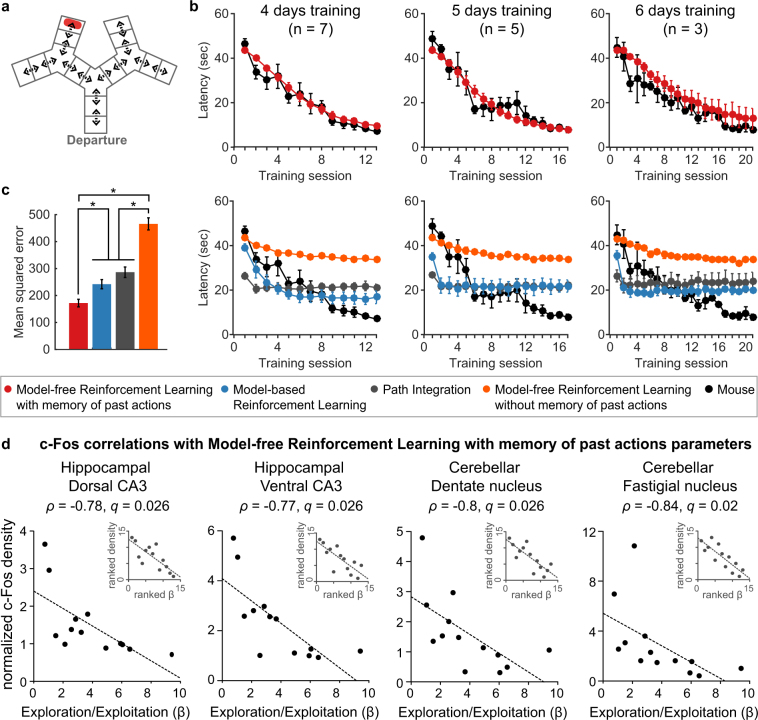
Neural correlates of the exploration-exploitation balance of model-free reinforcement learning model with a memory of past actions. (**a**) Virtual maze used for the simulations. The maze was discretized in corridors, dead-ends and intersections. (**b**) Average curves of simulations for each mouse, which reached the exploitation criteria in 4 days (left), 5 days (centre) or 6 days (right). The simulations with 100 freely choosing agents per mouse tested the ability of each model to reproduce the behavioural data (black). The optimal parameters used for the simulations were identified by fitting each mouse’s actions on a trial-by-trial basis (Supplementary Table 5). Only the model-free reinforcement learning model with a memory of past actions successfully replicated mice behaviour (top), whereas other models were unable to reach the mice’s final performances (bottom). (**c**) Average mean-squared error between the simulations and the mice’s performances for each model tested, showing significantly lower mean-squared error with the model-free reinforcement learning model with a memory of past actions (*indicates *q* < 0.05, FDR corrected, Mann Whitney comparisons). (**d**) c-Fos correlations with model-free reinforcement learning with memory of past actions exploration/exploitation trade-off, for the 13 mice which showed a higher log-likelihood amongst the three models which can learn the sequence. Only hippocampal and cerebellar structures showed significant correlations (*q* < 0.05, FDR corrected, Spearman correlation). The main plot shows the correlation on the raw data and the inset in the top right hand side shows the same correlation on the ranked data, which is used to calculate Spearman’s correlation. Data represent mean ± s.e.m.

We then simulated 100 iterations of each learning model with those optimal parameters (Fig. 3b, Supplementary Fig. 8a) and compared the mean latency to reach the platform from the 100 simulations with the mice’s learning curve by computing the mean-squared error between the two (Fig. 3c, Supplementary Fig. 8b).

This analysis first confirmed that the classical model-free reinforcement algorithm was unable to learn the correct sequence (Fig. 3b, orange curves, Supplementary Fig. 8a). With this algorithm indeed, intersections could not be distinguished and were thus all associated with the same action, making the algorithm unable to learn a sequence of alternating turns.

The path integration and model-based learning algorithms, which could learn the correct sequence, showed poor ability at reproducing the mice’s learning curve although using the set of optimally fit individual parameters (Fig. 3b, grey and blue curves, Supplementary Fig. 8a). The simulations using the optimal parameters of the model-free reinforcement learning algorithm with a memory of past actions was closest to the actual data (Fig. 3b, red, Supplementary Fig. 8a). Accordingly, the simulations with this algorithm had a significantly lower mean-squared error (Mann-Whitney FDR corrected, *q* = 2.03 × 10^−5^ for model-free RL with vs. without memory, *q* = 2.4 × 10^−4^ for model-free RL with memory vs. path integration, *q* = 5.1 × 10^−3^ for model-free RL with memory vs. model-based RL, while *q* = 0.07 for model-based RL vs. path integration, Fig. 3c and Supplementary Fig. 8b).

Considering the three models that were able to learn the correct path (path integration, model-based reinforcement learning, and model-free reinforcement learning with memory of past actions), the likelihood of each model to reproduce the mice’s decisions during learning favoured the model-free algorithm in 13 mice out of 15 mice (Supplementary Table 5a). We thus examined the covariance between structures’ c-Fos positive cell density obtained at the exploitation stage and the parameters of the model in the 13 mice for which the behaviour was best explained by this model, thus excluding mice 14 and 15. Note that, extracted at the individual level, learning rate (η), exploration/exploitation trade-off (β) and discount factor (γ) values in the model are constant across learning and characterize the whole learning curve of a given mouse.

The discount factor did not vary much across individuals (Supplementary Table 5a) and therefore, expectedly, did not covary with the activity of any structure (Supplementary Table 6b). The learning rate and the exploration/exploitation trade-off were negatively correlated (Spearman’s correlation, p < 0.01; Supplementary Table 6a): a high learning rate (high η) was associated with a higher tendency to explore new actions (low β) and a low learning rate (low η) was associated with a higher tendency to exploit knowledge about rewarding actions (high β).

Only hippocampal and cerebellar structures revealed correlated activity with the exploration/exploitation trade-off (β). Four structures had significantly negatively correlated c-Fos expression: dCA3, vCA3, dentate and fastigial nuclei (Spearman’s correlation, *q* < 0.05, FDR corrected; Fig. 3d, Supplementary Table 6b). Thus, the higher c-Fos density was in these four structures, the more the balance was toward exploration.

Furthermore, there was a tendency for positive correlations between the learning rate (η) and hippocampal and cerebellar c-Fos expression, involving the dCA1, vCA3, lobules IV/V, Crus I and fastigial nucleus (Spearman’s correlation*, q* < 0.07, FDR corrected; Supplementary Fig. 9, Supplementary Table 6b). That is, the higher the learning rate was, the higher number of cells expressed c-Fos during the final training session, and among these structures, dCA1 and lobules IV/V were the two hubs of the exploitation network. We used non-parametric correlation analysis, which calculates the correlations on ranked data points rather than values, thus ensuring that the correlations were not driven by two mice both showing particularly high learning rates and high c-Fos densities in the correlated regions (see insets in Fig. 3d and Supplementary Fig. 9).

In summary, amongst common navigation learning models, a model-free reinforcement learning model with a memory of past actions was able to reproduce the exploitation mice’s behaviour. At the level of individual mice, the exploration/exploitation trade-off of this model correlated with the c-Fos densities of hippocampal and cerebellar regions only. As c-Fos activity was extracted at a point where all mice had similar performance, this correlation analysis with learning parameters is orthogonal to the graph analysis performed during the exploitation stage. The two analyses thus suggest that hippocampal and cerebellar regions are not only central to the network of mice performing of sequence-based navigation but may also contribute to the dynamics of acquisition of this behaviour.

## Discussion

This study explores the functional networks underlying exploration versus acquisition and exploitation of sequence-based goal-directed navigation. To address this question we developed a navigation task constraining mice to use self-motion based information, and used a non-targeted analysis of brain regions’ activation and inter-regional correlations. We thus found that whereas large interactions between cerebral cortices and striatum underlie exploration, the exploitation of sequence-based memory is supported by a network centred around the hippocampus and cerebellum. The computational analysis further showed that the acquisition of this behaviour can be modelled by an algorithm combining a memory of past actions and model-free reinforcement learning processes, which parameters are correlated to activity in hippocampal and cerebellar regions.

Exploration and sequence-based memory exploitation are supported by two distinct major functional organization of the brain network: while the exploration phase is characterized by a cortico-basal ganglia cluster, possibly organized around the DMS as a hub^16^, the network underlying sequence-based navigation appears hippocampo-cerebellar centred.

In addition to hippocampal and cerebellar regions, the exploration and exploitation stages of sequence-based navigation engage widespread activations of structures classically involved in both navigation (e.g. retrosplenial and parietal cortices, associated with reference frame manipulation^43–46^ or egocentric motion coding^47–50^) and decision-making (medial prefrontal cortex and striatum^24,51,52^).

Familiar routes have been proposed to be learned as chains of stimulus-response-stimulus associations^53–56^, either through model-free reinforcement learning or on the basis of model-based reinforcement learning algorithms with a representation of the maze (‘world model’, but not necessarily allocentric) stored in the hippocampus^32,33^. Our data suggest that the acquisition of sequence-based navigation in an environment lacking unambiguous landmarks follows a model-free learning algorithm associated with a memory of past actions. This result is consistent with a theoretical study showing the beneficial contribution of a short-term memory buffer to model-free reinforcement to solve several spatial tasks otherwise unsolvable with standard model-free reinforcement learning models^57^. Model-based reinforcement learning (as well as path integration) can exploit information about the location of the reward as soon as it has been visited once, from any place of the maze (including the starting point), which is incompatible with the relatively slow learning dynamics observed here. Contrarily, the model-free reinforcement learning model requires multiple successful trials for the information about the reward to propagate and affect value estimation at the starting position. Consequently only the model-free reinforcement learning model was able to reproduce the slow increase in mice’s performance.

These results have two implications. First, the planning of future actions, which constitutes the basis of model-based reinforcement learning, does not appear as a necessary process to sustain the learning rules of sequence-based navigation. Second, the learning algorithm does not require a world model (or cognitive map) of the whole maze, thus questioning the role of the hippocampus in this acquisition. Instead, the learning algorithm requires maintaining the memory of past actions, a process potentially supported by the hippocampus.

Indeed, convergent evidence point toward a central role of the cerebellum and the hippocampus at the core of the processes allowing to learn and perform goal-directed sequence-based navigation from self-motion information.

In the exploitation phase, hippocampal and cerebellar regions have strongly correlated activity (see the high confidence network in Supplementary Fig. 4b) and they appear as hubs of the exploitation network. Two cerebellar clusters appear functionally connected with the dCA1 of the hippocampus. The first cluster involves lobule IV-V, lobule VI and Crus I. It is noteworthy that c-Fos activity is reported as a ratio between exploitation and motor control, therefore suggesting that the activation of this cluster is not due to motor execution only^58^ (but see in monkeys^59^ and in humans^60^). Cerebellar Crus I - hippocampal coupling was previously revealed during the execution of sequence-based navigation after learning the task in an environment rich in external landmarks^28^. Here we further reveal the involvement of the hippocampus when the task is performed in an environment lacking unambiguous landmarks and based on self-motion information. This study also points towards the implication of a second cerebellum cluster including lobules IX and X, which suggests that vestibular information processing performed by these cerebellar lobules^61,62^ may be of particular importance when performing sequence-based navigation, once the sequence has been learned.

Although we showed that the acquisition of the behaviour reported here is best modelled by a model-free reinforcement learning algorithm, the learning parameters neither correlated with cortical or basal ganglia areas, frequently associated with model-free reinforcement learning^34,37,63^. Instead, it was the activity of several hippocampal and cerebellar areas that correlated with the mice’s exploration/exploitation balance, and potentially learning rate. Although the cerebellum is usually associated to supervised learning^34,64^, reward prediction signals have recently been identified in the inferior olive^36^ as well as in the granule cells of the cerebellum cortex^65^ in stimulus-response tasks. These signals, similar to the reward prediction error central to model-free reinforcement learning algorithms, together with our results, suggest that the cerebellum could participate in model-free reinforcement learning in a sequence-based navigation task along with the hippocampus.

While hippocampal and cerebellar regions show correlated activity, the directionality of their interaction cannot be deduced from c-Fos analysis, and two interpretations can be considered.

First, although experimental proof of such directionality has not been reported yet, we cannot exclude that the hippocampus activity influences the cerebellum. The correlation between dCA1 and cerebellar cortices, which receive the main inputs entering the cerebellum, could suggest a connection from dCA1 to cerebellum. The hippocampus (dCA1) would implement the internal representation of the contextual information mandatory to perform the task (i.e. a combination of actual state and past actions). Under the light of the studies showing error prediction signals in the cerebellum^36,65^, the cerebellum could be the learning module in our task, performing reinforcement learning on contextual information provided by hippocampal inputs.

Conversely, it has been recently shown that hippocampal activity can be triggered by self-motion signals in the absence of external landmarks^20^. Here, the dCA3, which has been proposed to store sequences of events^66,67^ and convey memory-based representations to CA1^68,69^, has increased activity in the exploitation phase. It also belongs to a cluster including the parietal and retrosplenial navigation-related cortices^47,50,70,71^, known to provide information about the mouse’s position and orientation in space. That the medial entorhinal cortex had neither increased c-Fos activation nor correlated activity within the network of exploitation mice further points toward a greater importance of memory-based inputs in sequence acquisition, compared to spatial context inputs, which are usually conveyed via the MEC^14,72^. Thus the dCA3 could store the memory of past actions required by our model. Interestingly, the activity of the cerebellar fastigial nucleus, an output of the cerebellum that may project to the hippocampus^73–75^, increased during the exploitation phase and correlated activity with the exploration/exploitation trade-off, suggests that the acquisition and exploitation of sequence-based memory could depend on the cerebellum sending output signals to other activated brain regions. Hence our results are compatible with the cerebellum providing the hippocampus with self-motion signals to be memorized through CA3.

In summary, the hippocampus could support a memory of past actions to distinguish intersections in sequence-based navigation, which would conform with its proposed role in the temporal organization of events^76^, while the cerebellum could integrate self-motion information to allow the hippocampus update those past actions^77,78^ and/or support learning of the sequence taking into account contextual information from the hippocampus.

Our findings suggest that the emergence of goal-directed actions from the spatio-temporal organization of self-motion information builds upon a network where the hippocampus and the cerebellum occupy a central position, highlighting how hippocampal-dependent memory can be functionally associated with cerebellum activity beyond its motor aspect. This work is also proposed to serve as a work model to further test causal implication of the identified structures in exploration and goal-directed navigation.

## Materials and Methods

### Behavioural study

All experimental procedures were carried out in accordance with international (European parliament Directive 2010/63/UE) and national legislations (Decree no. 2013-118) on laboratory animals’ care and use and were approved by the Animal Ethics Committee of University Paris-6 (no. 00899.01). Ninety-nine male C57BL/6 J mice (12–14 weeks old) from Janvier (France) were used in this study, divided in five groups: exploration (n = 15), exploitation (n = 30), exploration swimming control (n = 15), exploitation swimming control (n = 15) and cage control (n = 24). They were housed in groups of 5 in standard conditions: 12 h light/dark cycle, with ad libitum access to water and food. Seven days prior to the beginning of behavioural tests, mice were separated and housed individually to limit inter-boxes variability resulting from social relationships. All behavioural experiments took place during the light cycle.

Prior training for sequence-based navigation, mice performed a series of S.H.I.R.P.A. protocol tests^79^ to ensure their general good health and motor performance and habituate them to being manipulated. The tests included general observations (appearance, spontaneous behaviour and neurological reflexes), anxiety and motor abilities tests (motor coordination, balance, rotarod and muscular strength tests) and were performed within five days (details in^80^).

Sequence-based navigation was performed in a triple y-maze composed of a central stem from which two y-mazes originate (Fig. 1a). Alleys are 41 cm long, 25 cm wide and filled with water made opaque with an inert nontoxic product (Accuscan OP 301, Brenntage, Lyon, France) to hide a platform 1 cm below the surface. Water temperature was maintained between 19 and 21°C. White noise (50–60 dB) was used to cover sounds that the mice could have used to orientate themselves. A round black curtain without cues surrounded the maze and was turned every 4 trials to prevent the use of distal landmarks. The departure and arrival points were fixed so that the fastest/shortest way to reach the platform was to swim a left then right sequence of turns. As all three intersections of the maze were visually identical, mice had to remember the sequence of turns to solve the task. Trials lasted up to 60 sec. When trials were unsuccessful, mice were placed back into the departure alley and guided to the platform. Intertrial and intersession intervals were 30 s and 45 min respectively.

Exploration mice performed one session of four trials in the triple-y maze. To avoid c-Fos expression related to discovering the features of the task (such as swimming), they did a pre-training session the previous day in which they swam four trials (60 sec each) in a two-alley corridor with no platform. Exploitation mice were trained up to six days doing four sessions of four trials per day. To ensure equivalent task mastery amongst them for the c-Fos network analysis, mice had to perform 75% of correct trials during one training day and one 100% correct session the following day.

We had two control groups aimed at matching the exploration group and the exploitation group, for the stress and motor activity induced by the task (respectively ‘exploration swimming control’ and ‘exploitation swimming control’). Each control mouse was randomly paired with an experimental mouse and followed the same training schedule as their paired mouse, swimming the same duration in a two-alley corridor. No escape platform was present so the animal was removed from the pool by the experimenter when the trial ended. One control mouse (paired with an exploitation mouse) that jumped out of the maze instead of swimming on the final day was excluded from the study.

Cage control mice were used to measure baseline expression of c-Fos. They did not perform the task but were brought to the experimental room and handled daily by the experimenter, similarly to the four other groups.

Data acquisition was performed by means of a video recording system and tracking software, SMART (BIOSEB, Chaville, France) and data processing was automated via a MATLAB batch program developed in our laboratory (Navigation Analysis Tool^81^).

### c-Fos immunohistochemistry

Ninety minutes after the last trial, mice were deeply anesthetized with an intraperitoneal injection of ketamine (150 mg/kg) and xylazine (12 mg/kg) and perfused transcardially with saline (0.9%), followed by an ice-cold solution of 4% paraformaldehyde in phosphate buffer (PB 0.1 M, pH 7.4). After postfixation overnight in the same fixative at 4 °C, brains were cryoprotected for 48 hours in a sucrose solution (30% in PB 0.1 M, pH7.4) at 4 °C. 50 µm-thick coronal sections were cut on a freezing microtome and stored in PB 0.1 M solution containing 0.02% of sodium azide.

Free floating sections were rinsed in 0.1 M PB and incubated for 30 min with H_2_O_2_ hydrogen peroxide (0.3% in PB). After PB rinses, sections were incubated overnight with anti-c-Fos primary rabbit polyclonal antibody (1:1000, Santa Cruz) diluted in blocking solution (PB 0.1 M, 0.1% BSA, goat serum 2%, 0.2% Triton X-100). A biotinylated goat anti-rabbit antibody (1:500, Jackson Immunoresearch) was used as secondary antibody. After washing, staining was revealed using the avidin-biotin peroxidase method (ABC kit, Vectastain Elite kit, Vector Laboratories, Burlingame, CA, USA) coupled to diaminobenzidine as a chromogen. Sections were mounted on gelatin-coated slides.

#### c-Fos positive cell quantification

Quantitative analyses of c-Fos positive cell were performed using a computerized image processing system (Mercator, Exploranova, La Rochelle) coupled to an optical microscope. The quantification of c-Fos positive cell was carried out at 10x or 20x magnification. Structures were defined according to the Franklin and Paxinos atlas^82^ and Allen Brain Institute Atlas for cerebellar regions^83^. Immunoreactive neurons were counted bilaterally using three consecutive sections spaced 200 µm apart. Quantifications were conducted ‘blind’ with respect to assignment of mice to groups.

c-Fos positive nuclei densities were quantified in 34 area likely involved in goal-directed navigation processes. These included cortices: prelimbic, infralimbic, cingulate 1 and 2, dysgranular and granular retrosplenial, parietal and posterior parietal, medial entorhinal; striatal regions: dorsomedial and dorsolateral striatum, nucleus accumbens core and shell; dopaminergic nuclei: ventral tegmental area and substantia nigra pars compacta; hippocampal subfields: dorsal CA1, CA2, CA3 and dentate gyrus and ventral CA1, CA3 and dentate gyrus; cerebellar regions: lobules IV/V, VI, VII, IX, X, hemispheres Simplex, Crus I and II, dentate, fastigial and interpositus nuclei. The primary auditory cortex was counted as comparison as auditory information was not relevant for solving the task.

The immunohistochemical procedure was performed in two cohorts, each containing mice belonging to each experimental group. To avoid differences due to immunohistochemical procedures, raw cell counts were normalized within cohorts to that of either swimming control mice or cage control mice. In either case, the normalization was performed as follows: within a immunohistochemical cohort, each brain region’s raw count (mean number of c-Fos positive nuclei per mm² across three consecutive sections) for any given mouse was divided by the same region’s mean raw count of the control group (either swimming or cage). Homogeneity of normalized counts within experimental groups across cohorts was verified by Mann-Whitney U tests. Results were then averaged per group and expressed as a percentage.

Differences in normalized c-Fos positive cell density between experimental groups were assessed using Kruskal-Wallis for each structure and post-hoc comparisons were performed using the Mann-Whitney U test.

### Functional connectivity analysis

Pairwise correlations between normalized c-Fos positive cell densities in the 34 regions were determined by computing Spearman’s correlation. Each set of correlations was displayed as color-coded correlation matrices using R software (Fig. 2a; Supplementary Figures 6 and 7).

Networks graphs were constructed by considering positive correlations at three two-tailed significance levels, p < 0.05 corresponding to ρ ≥ 0.51, p < 0.01 corresponding to ρ ≥ 0.64, and p < 0.002 corresponding to ρ ≥ 0.73. Negative correlations (which may represent regional activity suppression) were not considered as they are not handled by the functional connectivity analysis used in this study^84^.

Hub structures were identified using two measures of centrality: degree and betweenness^38^, computed with the Brain Connectivity Toolbox (https://sites.google.com/site/bctnet/)^84^. Degree corresponds to the number of structures a structure is significantly correlated to. Betweenness is the number of short communication paths a structure participates in. All structures were ranked by degree and betweenness, and candidate hub structures were defined as the structures ranked >80^th^ percentile for both measures in all three confidence levels’ networks.

Cluster analysis were conducted on the networks thresholded at p < 0.01. The analysis was based on the Markov Cluster Algorithm (inflation parameter set at 2.1), a scalable, unsupervised cluster algorithm for networks based on simulation of stochastic flow in graphs^42^. The clustering and associated visualizations (graph networks, Fig. 2b and clusters, Fig. 2c) were performed using Cytoscape (http://www.cytoscape.org/).

### Computational learning study

We explored the most parsimonious learning models with the potential to explain the observed behaviour. We considered classical model-based and model-free types of reinforcement learning algorithms. Given the constraints of the task, we assumed that the simulated agents could not rely on allocentric place representations to navigate, but instead on the cruder available sensory and motor information. In such situation, strategies relying on path integration are also expected to be recruited; we thus also tested a Bayesian learning model using path integration to monitor the information of self-position with respect to the departure point.

The sensory inputs *I* encoded the local shape of the maze and could take three different values, respectively corresponding to corridors (I), dead-ends (u) and Y intersections (Y). Four actions were defined: forward (F), left (L), right (R) and U-turn (U). Only actions for which openings were available were considered.

#### Model-based reinforcement learning

Based on the sensory inputs {I, u, Y,}, the model-based reinforcement learning algorithm gradually built an internal representation (*model*) of the states encountered and of the transition probabilities between those states. This internal model was then used to plan the shortest path to the reward using search algorithms^85^. Because of the limited external cues and their ambiguity, many different positions in the maze generated the same sensory inputs but the states were disambiguated by their position in the graph (Supplementary Fig. 10). Note that with this algorithm, when an agent made a U-turn, it was not aware that it may go back to a previously experienced node (Supplementary Fig. 10c).

When beginning a trial, the agent considered it always started from the same node *N*_0_ (in brown in Supplementary Fig. 10). When the agent left any node *N*_*k*_ and tried an action *a*_*i*_ never tried before, then a new node *N*_*m*+1_ was added to the *m* currently known nodes. If the new node contained the platform, the information that reward was available in that node was stored $(R({N}_{m+1})=1)$, otherwise *R*(*N*_*m*+1_) was set to 0. A new transition was also created:

1 $$T({N}_{k},{a}_{i},{N}_{m+1})=\eta \,$$

Then, if the node *N*_*m*+1_ was explored again in a later trial (i.e. if the agent performed the same succession of actions), the transition probability that from node *N*_*k*_, action *a*_*i*_ led to node *N*_*m*+1_ was updated as follows:

2 $$T({N}_{k},{a}_{i},{N}_{m+1})\leftarrow T({N}_{k},{a}_{i},{N}_{m+1})+\eta (1-T({N}_{k},{a}_{i},{N}_{m+1}))\,$$

The parameter *η* was thus the learning rate of the transitions.

Using the current representation of the task (the set of nodes *N* and the set of transitions *T*), the agent could evaluate the value of choosing a given action in the current state. This was done by propagating the reward information within the graph to attribute to each state a value *V* depending on its distance to the rewarded states, using a constant discount factor *γ*. This propagation, known as value iteration, consisted in repeating the following operation over all nodes, until convergence:

3 $$V(N)\leftarrow max(R(N),V(N),{ma}{{x}}_{i}(\gamma T(N,{a}_{i},N^{\prime} )V(N^{\prime} )))\,$$

Action selection was performed by computing for the current node *N*_*k*_ the value *Q*(*N*_*k*_*, a*_*i*_) of each action *a*_*i*_, which was either 0 if the action had never been tried, or $\gamma T(N,{a}_{i},N^{\prime} )V(N^{\prime} )$, with *N*′ the node which was attained when choosing *a*_*i*_. The final selection was made by drawing in the probability distribution defined as a softmax of the Q-values:

4 $$P({N}_{k},{a}_{i})=\frac{{e}^{\beta Q({N}_{k},{a}_{i})}}{{\sum }_{j}{e}^{\beta Q({N}_{k},{a}_{j})}}$$

This model was thus characterized by three parameters:

1. η the learning rate of the transitions,
2. γ the discount factor of the value iteration process,
3. β the exploration/exploitation trade-off of the softmax action selection.

#### Model-free reinforcement learning

The model-free reinforcement learning algorithm used was the standard *actor-critic*, from the TD-learning family. Contrary to the model-based algorithm, there is no representation of the transitions between states to distinguish states associated with identical sensory inputs (e.g. the three intersections). To allow the algorithm to learn a different action at each intersection, we thus considered states *s* resulting from the concatenation of the sensory input *I*, with a memory of the *n* past performed actions:

5 $${s}^{t}=({I}^{t},{a}^{t-1},\ldots ,{a}^{t-n})\,$$

with *I*_*i*_ ∈ {I, u, Y} and *a*_*i*_ ∈ {F, L, R, U}.

We set the size of the action memory to t = 3, which corresponded to three consecutive actions in the discretized version of the maze and allowed maintaining a memory of the first intersection when arriving at the second intersection along the correct sequence to the platform, and thus disambiguate both intersections.

We also tested a size of 0, which corresponds to a memory-less model, as a control.

The critic component of the algorithm learnt to evaluate the value *V(s* ^*t*^) of a given state *s* ^*t*^, while the actor learnt to attribute the value *p(s* ^*t*^*, a)* of choosing action *a* in state *s* ^*t*^. The *V* and *p* values were then updated using the reward prediction error *δ*, where *r* ^*t*^ is the reward delivered at time *t*:

6 $${\delta }^{t}={r}^{t}+\gamma V({s}^{t+1})-V({s}^{t})\,$$

in the following manner:

7 $$V({s}^{t})\leftarrow V({s}^{t})+\eta {\delta }^{t}\,$$

8 $$p({s}^{t},{a}^{t})\leftarrow p({s}^{t},{a}^{t})+\eta {\delta }^{t}\,$$

Action selection was performed by drawing in the distribution resulting from a softmax applied to *p(s, a)*:

9 $$P(s,{a}_{i})=\frac{{e}^{\beta p(s,{a}_{i})}}{{\sum }_{j}{e}^{\beta p(s,{a}_{j})}}\,$$

This model was thus characterized by three parameters:

1. *γ* the discount factor on future reward value
2. *η* the learning rate of the actor and critic,
3. *β* the exploration/exploitation trade-off of the softmax action selection.

#### Path integration

Path integration maintained at every time step *t* an estimation of the position *P* ^*t*^(*Pos*) of the agent during its displacements with regards to its departure point. *P* ^*t*^(*Pos*) was modeled as a Gaussian 2D distribution, centered on the current position of the agent in the environment. To model the accumulation of errors intrinsic to path integration processes, its standard deviation increased at each time step:

10 $${P}^{t}(Pos)=N(\mu =(x(t),y(t)),\sigma ={\sigma }_{o}t)\,$$

The internal estimation of the position of the goal *P*(*Goal*) was initially a uniform distribution over the 2D space, which was updated as follows every time the goal was attained, using the current estimation of the position *P* ^*t*^(*Pos*):

11 $$P(Goal)\leftarrow (1-\eta )P(Goal)+\eta {P}^{t}(Pos)\,$$

Using these two distributions, a distribution of the directions *P* ^*t*^(*Dir*) leading most probably to the goal was computed for any direction *α*:

12 $${P}^{t}(Dir=\alpha )=\frac{{\sum }_{{D}^{\alpha }}{P}^{t}(Pos)\times P(Goal)}{{\sum }_{(Pos,Goal)}{P}^{t}(Pos)\times P(Goal)}\,$$

with

13 $${D}^{\alpha }=\{(Pos,Goal)|atan2(Goal-Pos)=\alpha \}\,$$

Action selection was performed by drawing in the distribution resulting from a softmax applied to *P* ^*t*^(*Dir*):

14 $${P}^{t}({a}_{i})=\frac{{e}^{\beta {P}^{t}(Dir={a}_{i})}}{{\sum }_{j}{e}^{\beta {P}^{t}(Dir={a}_{j})}}\,$$

This model was thus characterized by three parameters:

1. σ_*o*_, error accumulation in position estimation,
2. *η* the learning rate of the goal position estimation,
3. *β* the exploration/exploitation trade-off of the softmax action selection.

### Individual optimization and simulation procedure

We conducted trial-by-trial analyses to optimize parameters for each model and each mouse^86^. We discretized mice trajectories according to our model maze (Fig. 3a), so as to generate for each individual a sequence of decisions $D=\{{d}^{1},{d}^{2},\ldots ,{d}^{N}\}$ comparable to the decisions made by the three models. The optimization of the parameters to find the maximum likelihood $\hat{L}=\prod _{t}p({a}^{t}={d}^{t})$ was solved with the standard evolutionary algorithm NSGA-2 using the SFERES tool^87^. For this procedure, the probabilities of actions *p*(*a* ^*t*^) were computed for each model, but the action selection step was skipped, as the models were forced to make the same decision as the mouse (*d* ^*t*^). To avoid local optimum, we associated a diversity fitness function to ensure that the space of parameters was fully explored^88^. For each exploitation mouse, a pool of individuals representing different parameterizations of the same model was evolved during 200 generations, which ensured reaching stable likelihood values. This was repeated 10 times to prevent biases due to the random initial parameterizations. Since very similar optimized parameters were obtained across the 10 runs, the results from all 10 runs were pooled. The SFERES tool sampled parameters from a given range that we defined:

1. for the model-based reinforcement learning, the bounds were set as:
  η: [0.0, 1.0];
  β: [0.0, 200.0];
  γ: [0.0, 1.0];
2. for the model-free reinforcement learning, the bounds were set as:
  η: [0.0, 1.0];
  β: [0.0, 200.0];
  γ: [0.0, 1.0];
3. for the path integration, the bounds were set as:

η: [0.001, 1.0];

β: [0.0, 200.0];

σ_*o*_: [0.0, 1.0].

After parameter optimization, the quality of the fit for each individual was checked by simulating the behaviour of 100 freely choosing agents with the same parameter set, and comparing it to the behaviour of the corresponding mouse^89^. At the beginning of each trial, the agent was placed at the starting position. The moving speed of the agent was set to 19.6 cm/s, corresponding to the mean speed of exploitation mice on their last session, with a simulation time step equivalent to 1.05 s. Identically to the protocol used for mice, if the agent was not able to reach the platform in 60 s (or 57 steps), it was automatically put back to the starting position and guided to the reward zone along the correct path. Reaching the goal freely or guided was rewarded by R = 1. Unrewarded actions lead to R = 0.

Pairwise correlations between normalized c-Fos densities and the model’s parameters were determined by computing Spearman’s correlation.

### Code availability

The models were programmed in Python using the numpy library. The code is available on ModelDB and github (https://github.com/gviejo/Fos).

### Statistical analysis

Statistical analyses were run using Statistica 10.0 (Statsoft) (except when otherwise mentioned). Normality was tested using Shapiro-Wilk test for normality. Parametric tests were used (t-test) on normal data and non-parametric tests were used (Kruskal-Wallis, Mann-Whitney U, Sign, Spearman) on non-normal data or when the group had less than five observations. Multiple comparisons were accounted for by correcting the p-values using the False Rate Discovery (FDR)^90^. FDR-corrected *q* values inferior to 0.05 were considered significant.

## Electronic supplementary material

Supplementary Material
