# Author Correction: A hippocampo-cerebellar centred network for the learning and execution of sequence-based navigation

**DOI:** 10.1038/s41598-019-56345-7

**Published:** 2019-12-20

**Authors:** Benedicte M. Babayan, Aurélie Watilliaux, Guillaume Viejo, Anne-Lise Paradis, Benoît Girard, Laure Rondi-Reig

**Affiliations:** 1 Sorbonne Universités, UPMC Univ Paris 06, INSERM, CNRS, Neurosciences Paris Seine - Institut de Biologie Paris Seine (NPS - IBPS), Cerebellum Navigation and Memory team (CeZaMe), 75005 Paris, France; 2 Sorbonne Universités, Université Pierre et Marie Curie (UPMC), CNRS UMR 7222, Institut des Systèmes Intelligents et de Robotique (ISIR), F-75005 Paris, France

Correction to: *Scientific Reports* 10.1038/s41598-017-18004-7, published online 19 December 2017

The Supplementary Figure file that accompanies this Article contains an error in Supplementary Figure S1, where the cerebellum panel contained an incorrect annotation for Bregma −6.00 mm. The correct Figure S1 appears below as Figure 1.

**Figure 1:**
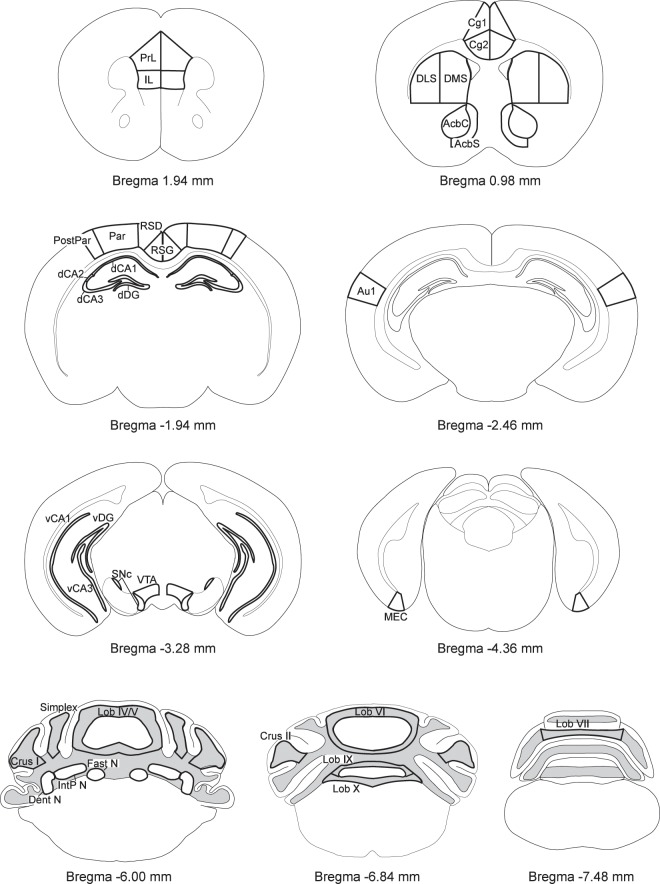
.

