# Supplementary material for: A hippocampo-cerebellar centred network for the learning and execution of sequence-based navigation
Source: Sci Rep. 2017 Dec 19;7:17812. doi: 10.1038/s41598-017-18004-7 (PMC5736633; doi:10.1038/s41598-017-18004-7)
Supplement: Supplementary file 1 — Supplementary Material [file 41598_2017_18004_MOESM1_ESM.pdf]

A hippocampo-cerebellar centred network for the learning and execution of sequence-based navigation

Benedicte M. Babayan[1], Aurélie Watilliaux[1], Guillaume Viejo[2], Anne-Lise Paradis[1], Benoît Girard[2] and Laure Rondi-Reig[1,]*

1- Sorbonne Universités, UPMC Univ Paris 06, INSERM, CNRS, Neurosciences Paris Seine - Institut de Biologie Paris Seine (NPS - IBPS), Cerebellum Navigation and Memory team (CeZaMe), 75005 Paris, France

2- Sorbonne Universités, Université Pierre et Marie Curie (UPMC), CNRS UMR 7222, Institut des Systèmes Intelligents et de Robotique (ISIR), F-75005, Paris, France

***Corresponding author:** Laure Rondi-Reig, laure.rondi-reig@upmc.fr

IBPS-Neurosciences Paris Seine, Sorbonne Universités, UPMC Univ Paris 06, CeZaMe team, 9 Quai Saint Bernard, 5e étage, 75005 Paris, France

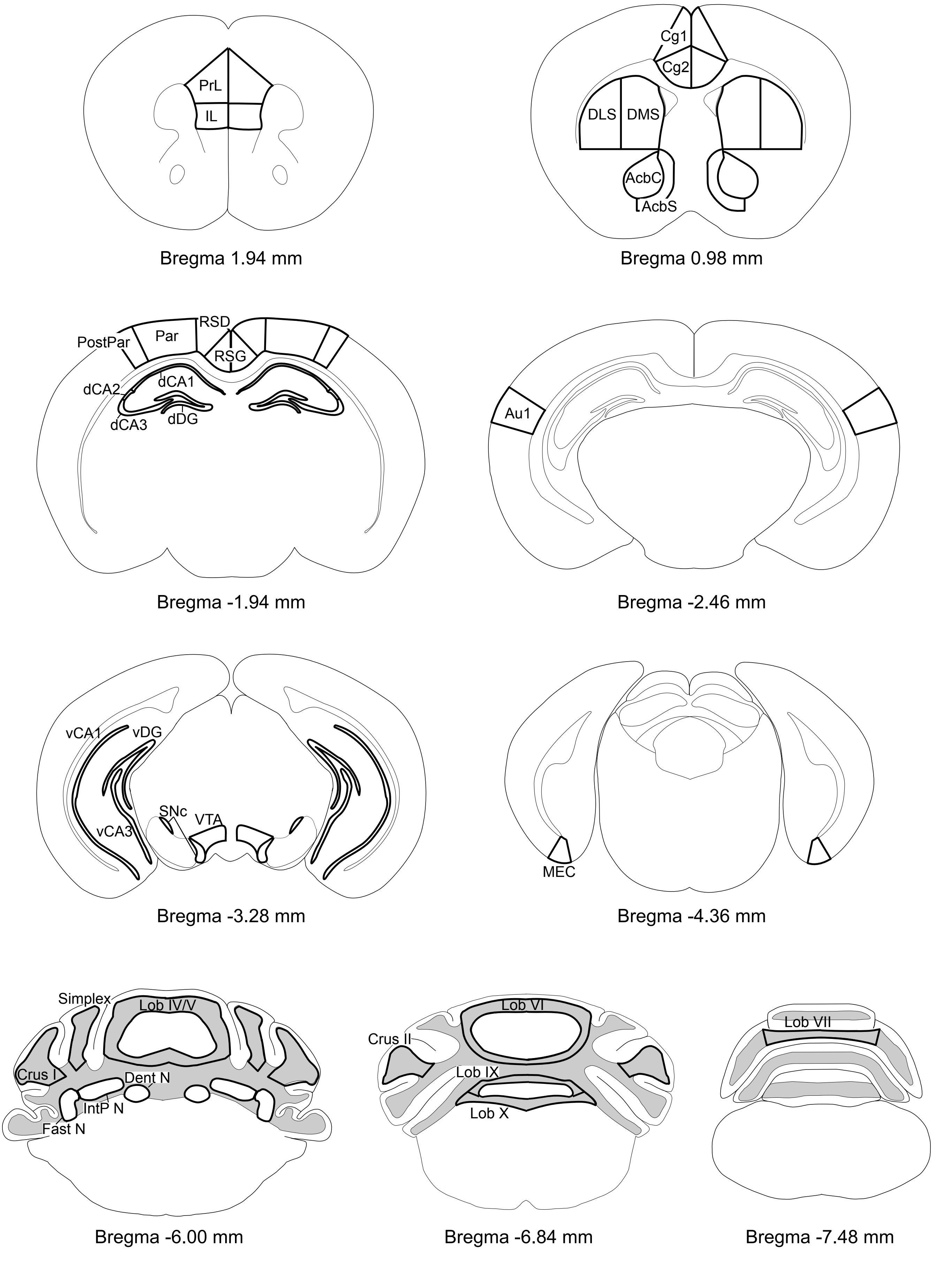

Supplementary Figure 1: Schematic representation of the structures analyzed for c-Fos expression.
Abbreviations: cortex: primary auditory (Au1), prelimbic (PrL), infralimbic (IL), cingulate 1 and 2 (Cg1, Cg2), dysgranular and granular retrosplenial (RSD, RSG), parietal and posterior parietal (Par, PostPar), medial entorhinal (MEC); striatum and dopaminergic nuclei (DA nuc): dorsomedial striatum (DMS), dorsolateral striatum (DLS), nucleus accumbens core (AcbC) and shell (AcbS), ventral tegmental area (VTA), substantia nigra pars compacta (SNc); hippocampus: dorsal CA1 (dCA1), dorsal CA3 (dCA3), ventral CA1 (vCA1), ventral CA3 (vCA3), dorsal CA2 (dCA2), dorsal and ventral dentate gyrus (dDG, vDG); cerebellum: lobules IV/V(Lob IV/V), VI (Lob VI), VII (Lob VII), IX (Lob IX), X (Lob X), dentate (Dent N), fastigial (Fast N) and interpositus nuclei (IntP N).

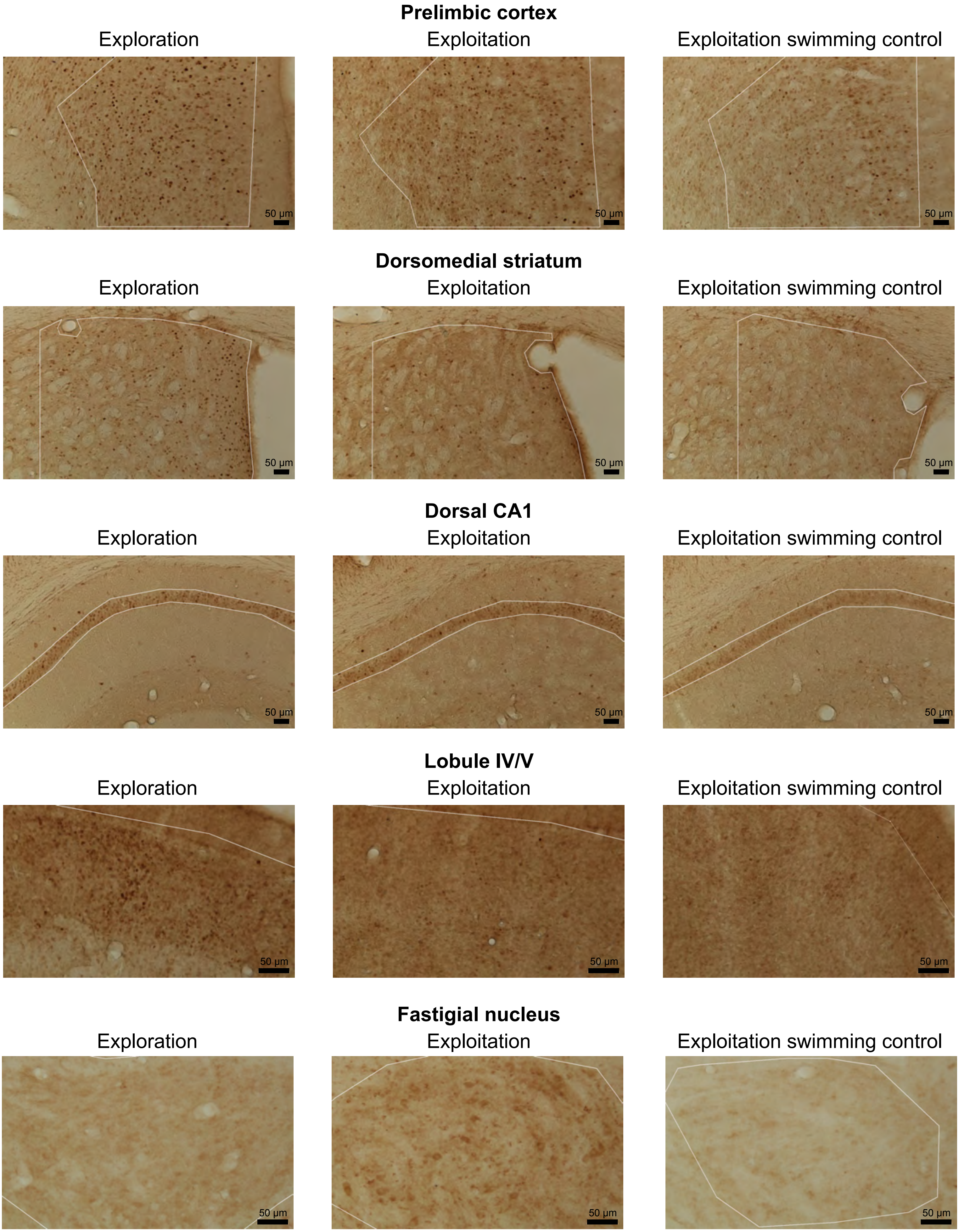

Supplementary Figure 2: Example c-Fos protein stainings in prelimbic cortex, dorsomedial striatum, dorsal CA1, lobule IV/V and fastigial nucleus. Stainings from mice belonging to exploration, exploitation and exploitation swimming control groups are shown. White lines correspond to the limits of the region counted, as shown in **Supplementary Figure 1**.

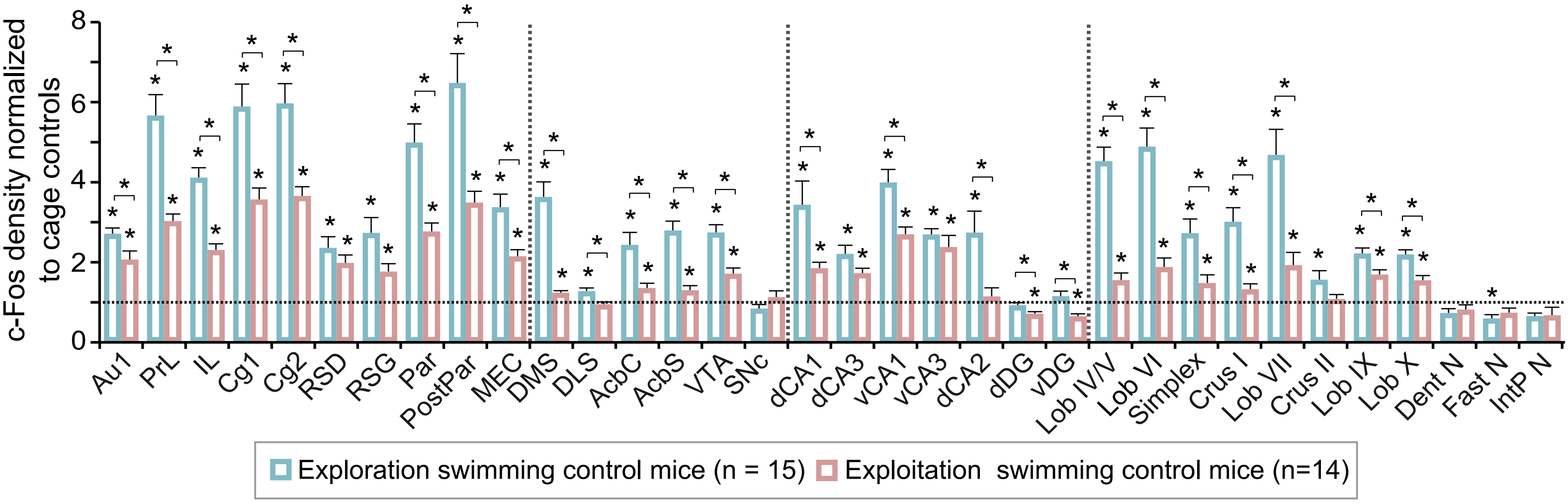

Supplementary Figure 3: Activation patterns across exploration and exploitation swiming controls normalized to cage controls. To compare c-Fos positive cell densities for exploration and exploitation swimming control mice, they are normalized to cage controls. Cage controls averages are indicated by the dotted horizontal line. * indicate significant differences ($q < 0.05$, FDR corrected, Mann Whitney comparisons). Data represents mean ± s.e.m.

Abbreviations: cortex: primary auditory (Au1), prelimbic (PrL), infralimbic (IL), cingulate 1 and 2 (Cg1, Cg2), dysgranular and granular retrosplenial (RSD, RSG), parietal and posterior parietal (Par, PostPar), medial entorhinal (MEC); striatum and dopaminergic nuclei (DA nuc): dorsomedial striatum (DMS), dorsolateral striatum (DLS), nucleus accumbens core (AcbC) and shell (AcbS), ventral tegmental area (VTA), substantia nigra pars compacta (SNc); hippocampus: dorsal CA1 (dCA1), dorsal CA3 (dCA3), ventral CA1 (vCA1), ventral CA3 (vCA3), dorsal CA2 (dCA2), dorsal and ventral dentate gyrus (dDG, vDG); cerebellum: lobules IV/V(Lob IV/V), VI (Lob VI), VII (Lob VII), IX (Lob IX), X (Lob X), dentate (Dent N), fastigial (Fast N) and interpositus nuclei (IntP N).

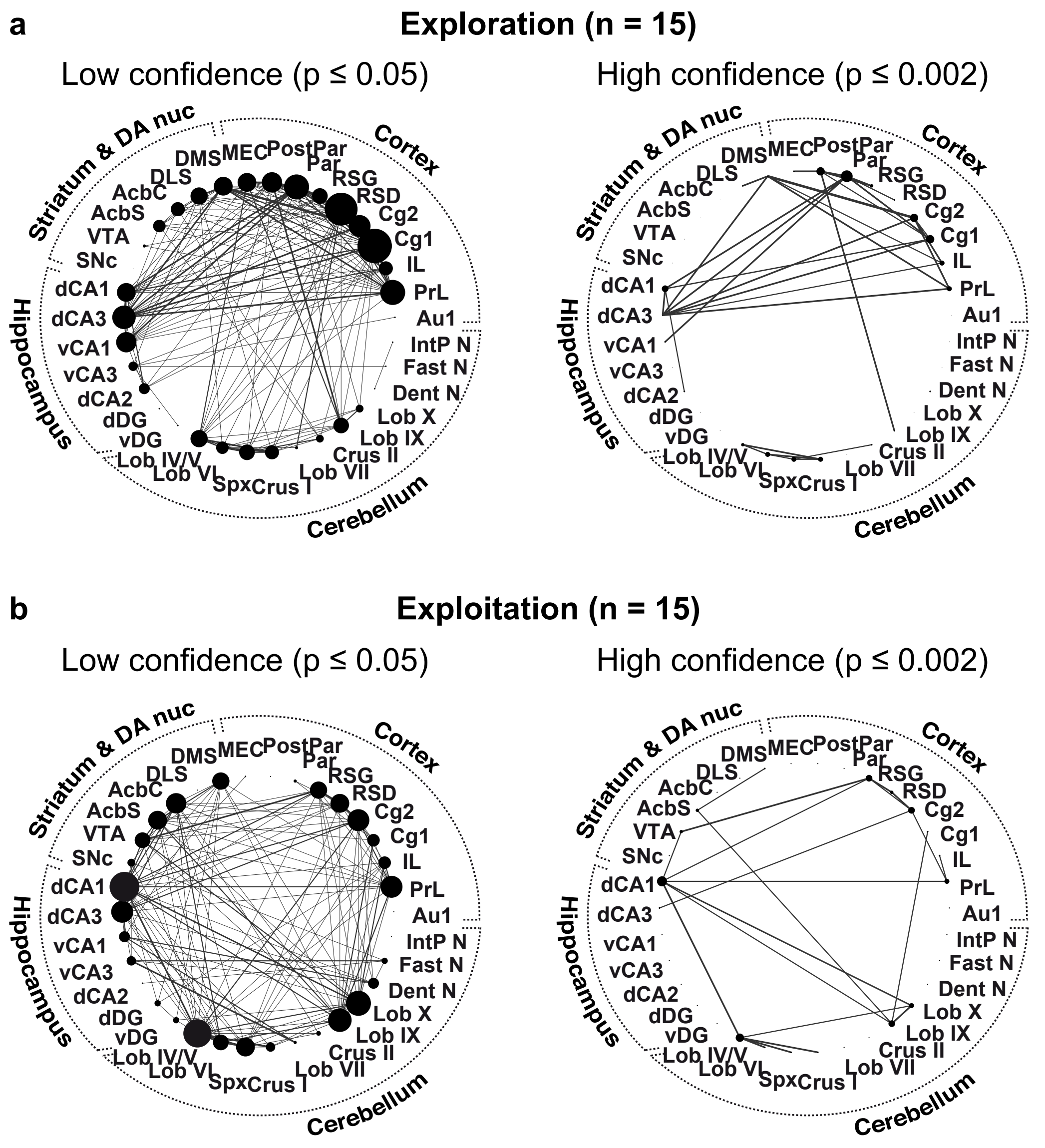

Supplementary Figure 4: Low and high confidence networks of sequence-based memory acquisition. Network graphs for exploration (**a**) and exploitation (**b**) mice normalized on exploration and exploitation swimming controls, respectively, were generated by thresholding inter-regional c-Fos correlations using a low confidence threshold (ρ ≥ 0.51, corresponding to a two-tailed significance level of p ≤ 0.05) or a high confidence threshold (ρ ≥ 0.73, corresponding to a two-tailed significance level of p ≤ 0.002). The main features observed at a p level inferior or equal to 0.01 were maintained across different thresholds. Abbreviations: cortex: primary auditory (Au1), prelimbic (PrL), infralimbic (IL), cingulate 1 and 2 (Cg1, Cg2), dysgranular and granular retrosplenial (RSD, RSG), parietal and posterior parietal (Par, PostPar), medial entorhinal (MEC); striatum and dopaminergic nuclei (DA nuc): dorsomedial striatum (DMS), dorsolateral striatum (DLS), nucleus accumbens core (AcbC) and shell (AcbS), ventral tegmental area (VTA), substantia nigra pars compacta (SNc); hippocampus: dorsal CA1 (dCA1), dorsal CA3 (dCA3), ventral CA1 (vCA1), ventral CA3 (vCA3), dorsal CA2 (dCA2), dorsal and ventral dentate gyrus (dDG, vDG); cerebellum: lobules IV/V (Lob IV/V), VI (Lob VI), VII (Lob VII), IX (Lob IX), X (Lob X), Simplex (Spx), dentate (Dent N), fastigial (Fast N) and interpositus nuclei (IntP N).

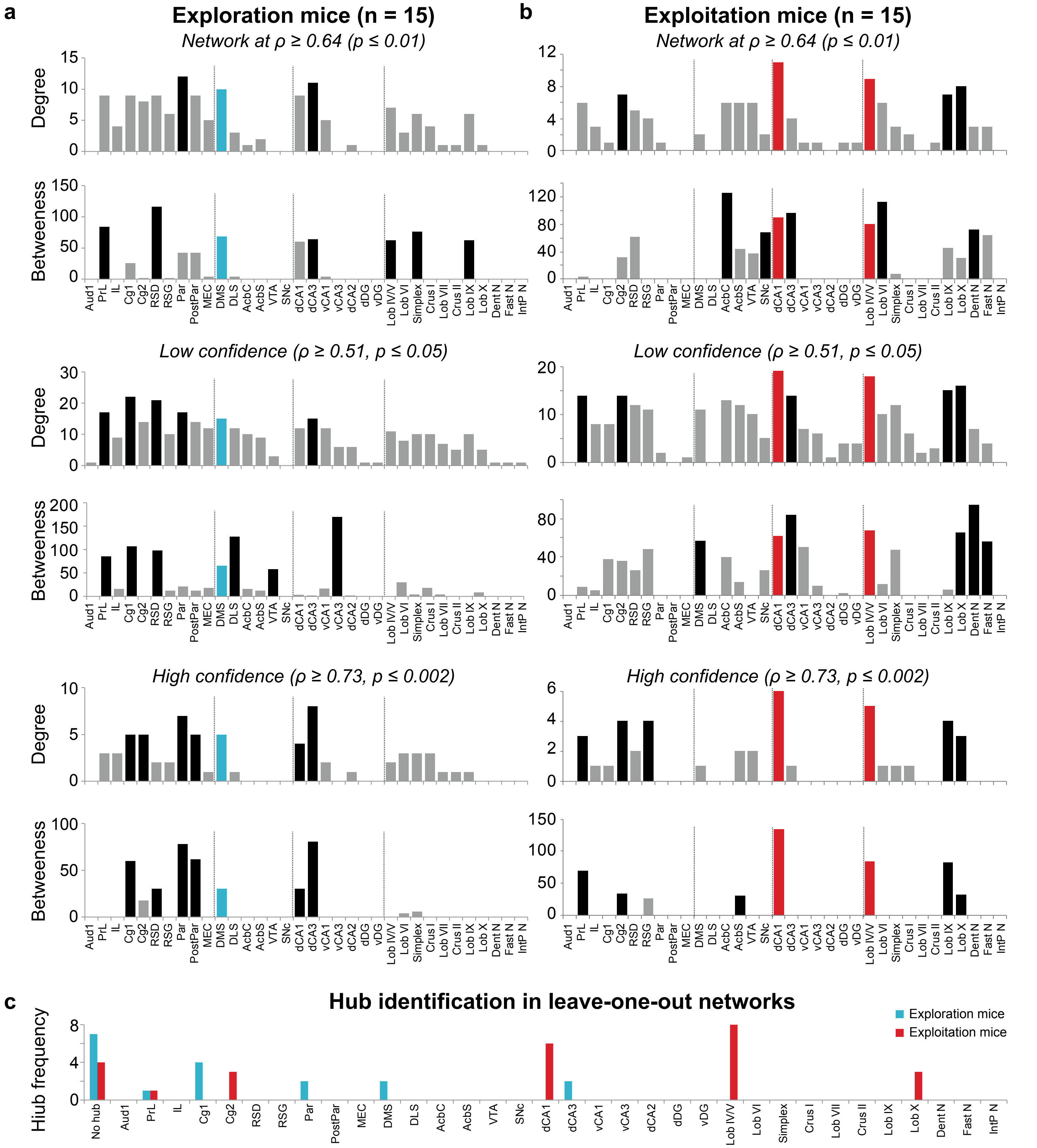

Supplementary Figure 5: Hub identification in exploration (a), exploitation (b) mice and 'leave-one-out' networks (c). Hub regions were identified as regions ranked above the 80th percentile for degree and betweenness across the three confidence levels' networks: $\rho \geq 0.64$, $\rho \geq 0.51$, $\rho \geq 0.73$. Regions above the 80th percentile within each confidence level are highlighted in black. Hub regions are identified in blue (**a**) and red (**b**) for exploration and exploitation mice, respectiv. In 'leave-one-out' analysis, a majority of exploration networks did not have hubs whereas dCA1 and lobule IV/V were the most often hub regions of exploitation leave-one-out networks (**c**).

Abbreviations: cortex: primary auditory (Au1), prelimbic (PrL), infralimbic (IL), cingulate 1 and 2 (Cg1, Cg2), dysgranular and granular retrosplenial (RSD, RSG), parietal and posterior parietal (Par, PostPar), medial entorhinal (MEC); striatum and dopaminergic nuclei (DA nuc): dorsomedial striatum (DMS), dorsolateral striatum (DLS), nucleus accumbens core (AcbC) and shell (AcbS), ventral tegmental area (VTA), substantia nigra pars compacta (SNc); hippocampus: dorsal CA1 (dCA1), dorsal CA3 (dCA3), ventral CA1 (vCA1), ventral CA3 (vCA3), dorsal CA2 (dCA2), dorsal and ventral dentate gyrus (dDG, vDG); cerebellum: lobules IV/V (Lob IV/V), VI (Lob VI), VII (Lob VII), IX (Lob IX), X (Lob X), dentate (Dent N), fastigial (Fast N) and interpositus nuclei (IntP N).

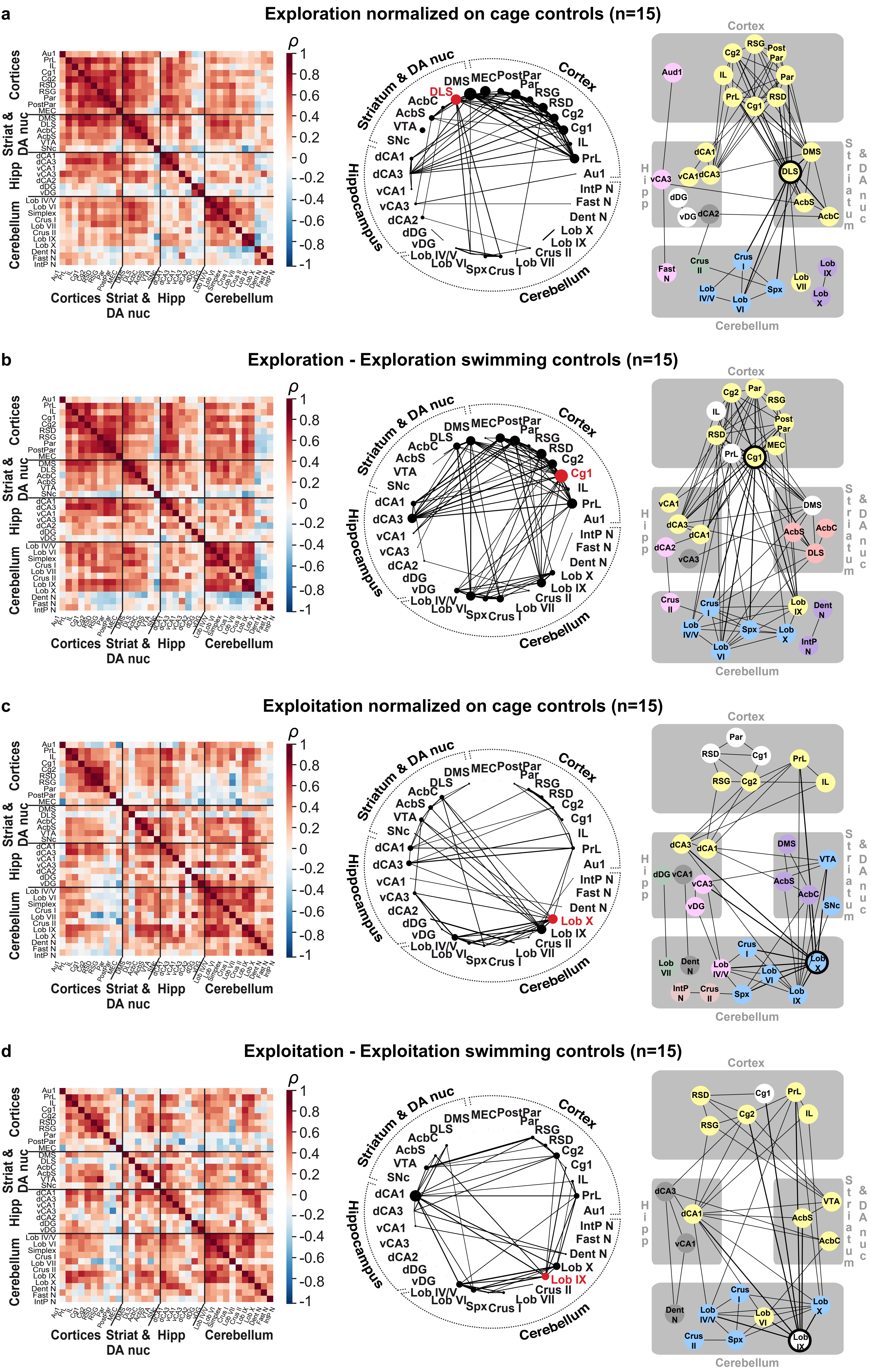

Supplementary Figure 6: Robustness of exploration and exploitation networks. The inter-regional correlations, hub and clustering analysis were performed on exploration and exploitation Fos densities normalized on cage controls (**a**, **c**) as well as on the substraction of exploration and exploitation Fos densities by their respective swimming controls (**b**, **d**). Left: Axes correspond to brain structures. Colors reflect correlation strength (scale, right). Middle: Network graphs considering the strongest correlations (Spearman's ρ ≥ 0.64, p ≤ 0.01), with the thickness of the connections proportional to correlation strength and node size proportional to degree. Right: Clusters are coded in different colors. Network hub structures are highlighted in red (middle) or black (right). The exploration mice's networks are centered around cortical correlations with striatal, hippocampal and cerebellar structures whereas hippocampo-cerebellar correlations are central in the exploitation networks, with cerebellar network hubs.

Abbreviations: cortex: primary auditory (Au1), prelimbic (PrL), infralimbic (IL), cingulate 1 and 2 (Cg1, Cg2), dysgranular and granular retrosplenial (RSD, RSG), parietal and posterior parietal (Par, PostPar), medial entorhinal (MEC); striatum and dopaminergic nuclei (DA nuc): dorsomedial striatum (DMS), dorsolateral striatum (DLS), nucleus accumbens core (AcbC) and shell (AcbS), ventral tegmental area (VTA), substantia nigra pars compacta (SNc); hippocampus: dorsal CA1 (dCA1), dorsal CA3 (dCA3), ventral CA1 (vCA1), ventral CA3 (vCA3), dorsal CA2 (dCA2), dorsal and ventral dentate gyrus (dDG, vDG); cerebellum: lobules IV/V (Lob IV/V), VI (Lob VI), VII (Lob VII), IX (Lob IX), X (Lob X), Simplex (Spx), dentate (Dent N), fastigial (Fast N) and interpositus nuclei (IntP N).

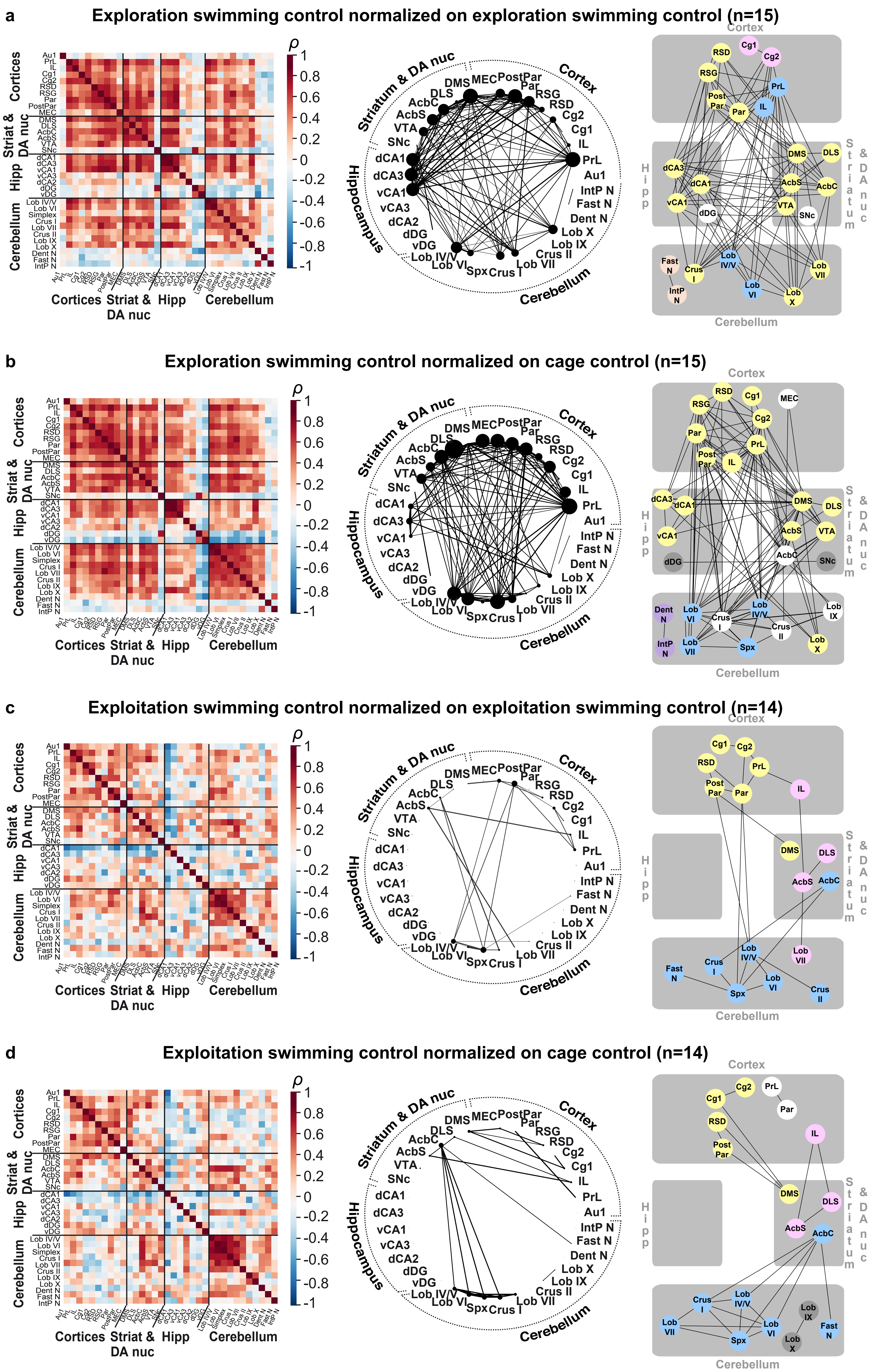

Supplementary Figure 7: Networks of exploration and exploitation swimming controls. The inter-regional correlations, hub and clustering analysis were performed on exploration and exploitation swimming controls Fos densities normalized on themselves (**a**, **c**) on cage controls (**b**, **d**). Left: Axes correspond to brain structures. Colors reflect correlation strength (scale, right). Middle: Network graphs considering the strongest correlations (Spearman's $\rho \geq 0.64$, $p \leq 0.01$), with the thickness of the connections proportional to correlation strength and node size proportional to degree. Right: Clusters are coded in different colors. The swimming exploration mice's networks are similar to exploration mice's network, but with more significant correlations at $p \leq 0.01$. Exploration swimming control network had sparse significant correlations, mostly within cortical and cerebellar regions. No regions in these networks met the hub criteria.

Abbreviations: cortex: primary auditory (Au1), prelimbic (PrL), infralimbic (IL), cingulate 1 and 2 (Cg1, Cg2), dysgranular and granular retrosplenial (RSD, RSG), parietal and posterior parietal (Par, PostPar), medial entorhinal (MEC); striatum and dopaminergic nuclei (DA nuc): dorsomedial striatum (DMS), dorsolateral striatum (DLS), nucleus accumbens core (AcbC) and shell (AcbS), ventral tegmental area (VTA), substantia nigra pars compacta (SNc); hippocampus: dorsal CA1 (dCA1), dorsal CA3 (dCA3), ventral CA1 (vCA1), ventral CA3 (vCA3), dorsal CA2 (dCA2), dorsal and ventral dentate gyrus (dDG, vDG); cerebellum: lobules IV/V (Lob IV/V), VI (Lob VI), VII (Lob VII), IX (Lob IX), X (Lob X), Simplex (Spx), dentate (Dent N), fastigial (Fast N) and interpositus nuclei (IntP N).

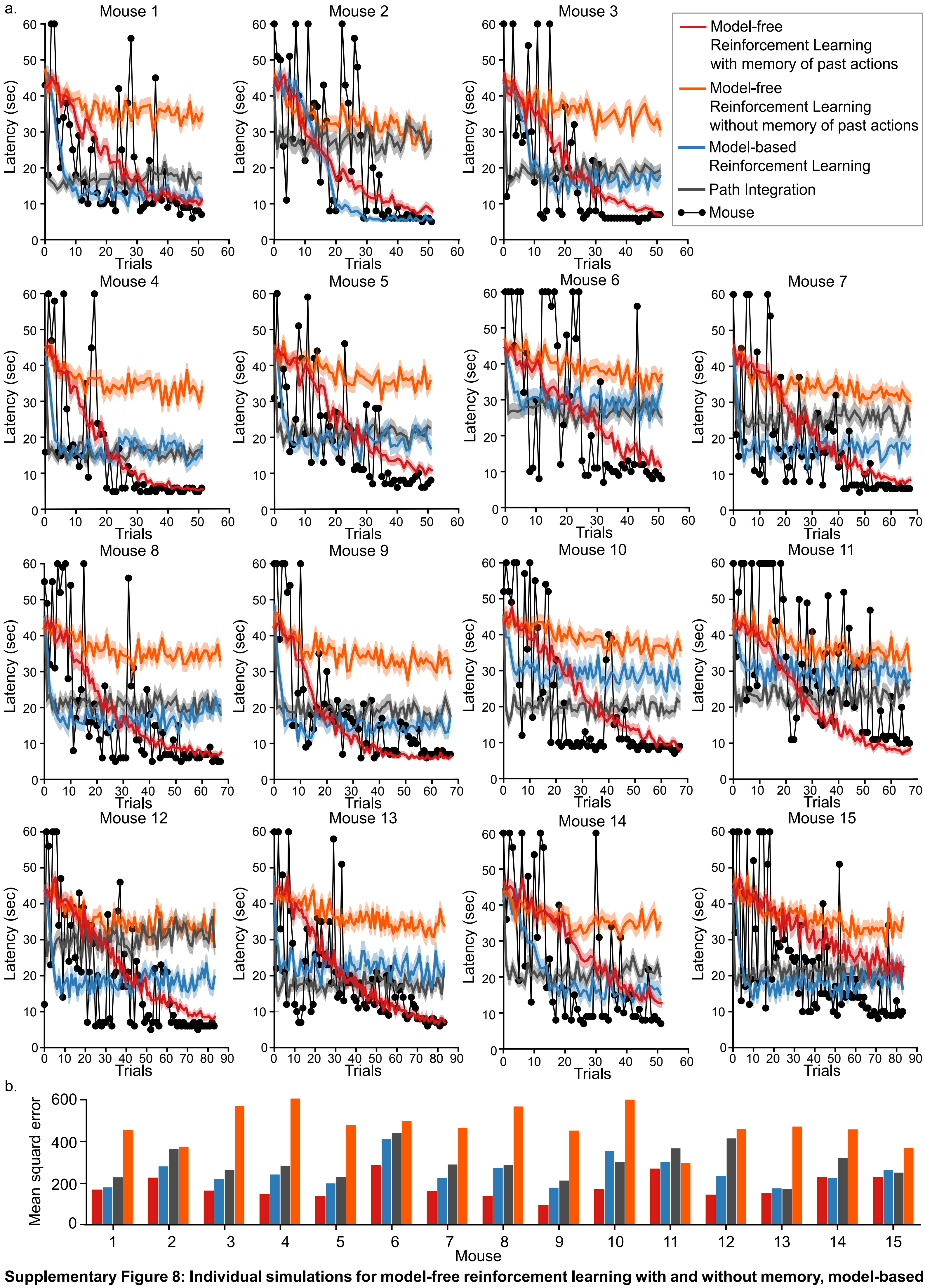

Supplementary Figure 8: Individual simulations for model-free reinforcement learning with and without memory, model-based and path integration models. (a) For each exploitation mouse, the optimal parameter set identified for model-free reinforcement learning with memory (red) or without memory (orange), model-based reinforcement learning (blue) and path integration (grey) was simulated on 100 freely choosing agents to test its ability to reproduce the behavioral data (black). Corresponding values for the parameters (η, the learning rate; β, the exploitation-exploration trade-off; γ, the discount factor for RL or σ the error on position estimation for path integration) are in **Supplementary Table 4**. Data represent mean ± s.e.m. (**b**) Mean-squared error (MSE) computed between observed behavior and model simulations using the best-fitting parameters identifying by log-likelihood analysis. Note that in all mice but one (14), the MSE is minimal for the model-free algorithm (in red).

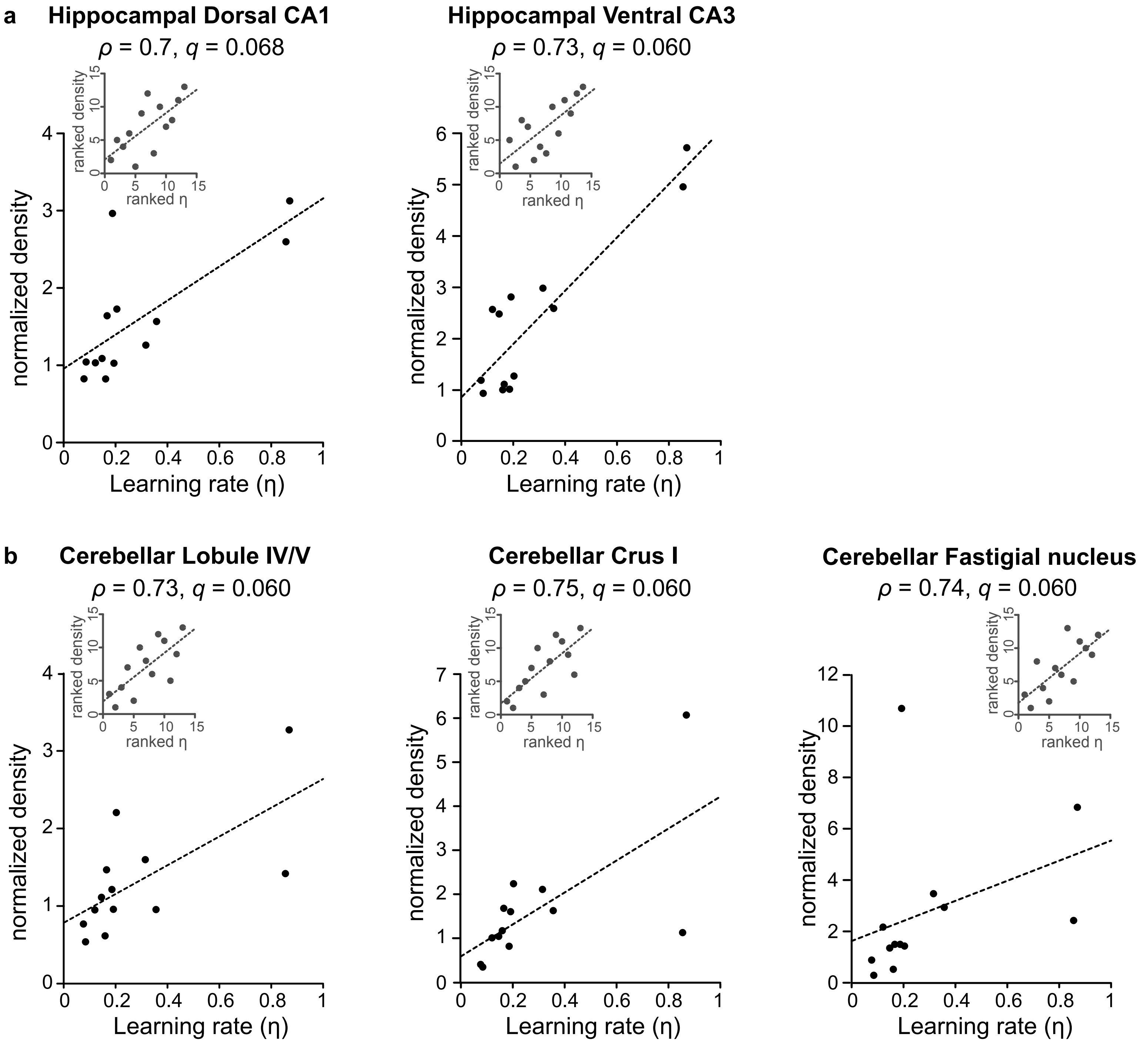

Supplementary Figure 9: Neural correlates of the learning rate. Hippocampal (a) and cerebellar (b) structures showed a tendency for correlations between c-Fos positive cell densities and individual learning rates estimated for the model-free reinforcement model with a memory of past actions. ($q < 0.07$, FDR corrected, Spearman correlation). The main plot shows the correlation on the raw data and the inset in the top right hand side shows the same correlation on the ranked data, which is used to calculate Spearman's correlation. Data represent mean ± s.e.m.

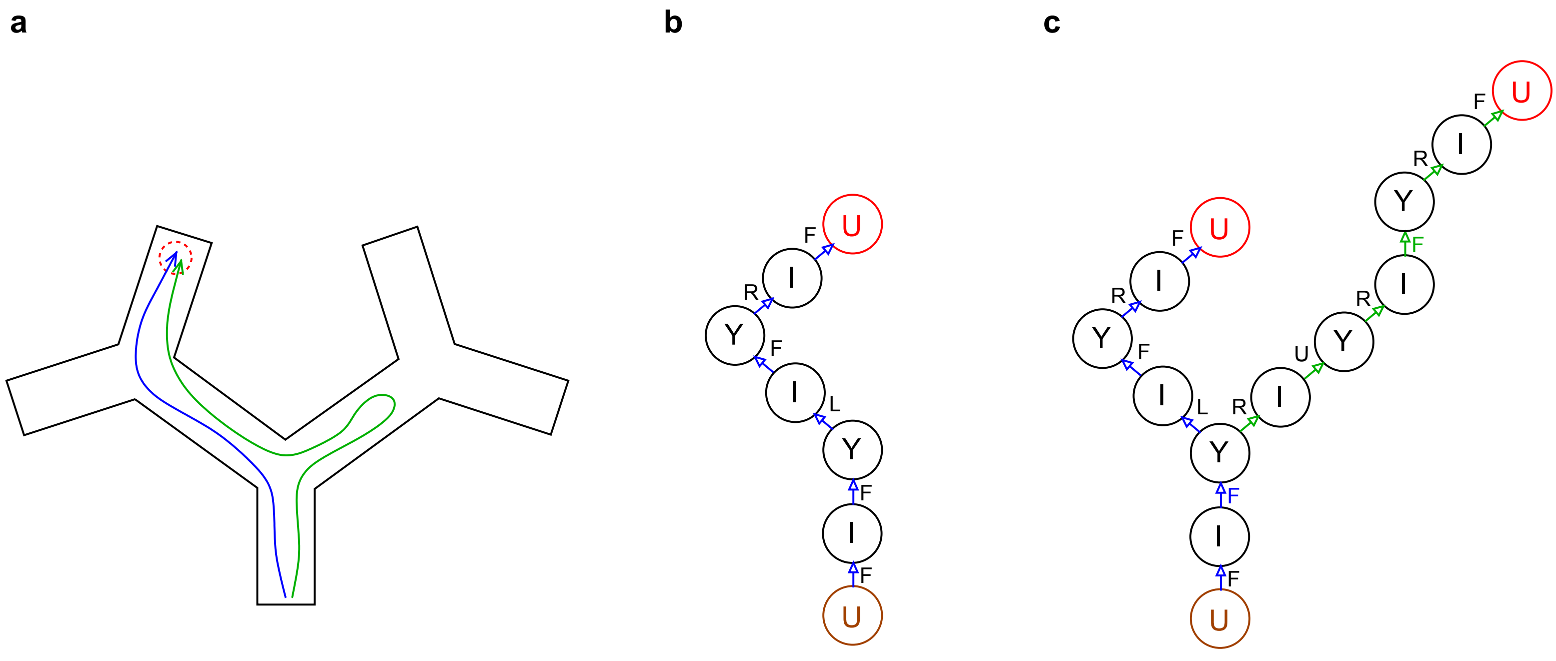

Supplementary Figure 10: Model-based algorithm internal representation. (**a**) Two trajectories experimented in the maze. (**b**) Internal graph built after the execution of the blue trajectory. (**c**) Updated graph after the execution of the green trajectory. The node in brown is the initial state, nodes in red are those where reward has been obtained (R=1), the color of transitions indicates during which trajectory they were created, and is strictly illustrative.
The states were based on sensory inputs only, they were thus ambiguous as many different positions in the maze generated the same perception. Still, the states were disambiguated by their position in the graph. For example, in the blue trajectory presented in (**a**) three identical corridors (I) were experienced and thus stored in the internal graph (**b**), the first one was reached by choosing the F action from the initial state, the second one was reached after a F-F-L sequence of actions, and the third one with the F-F-L-F-R sequence. Note that no backtracking memory was implemented, so that, when one agent made a U-turn, it was not aware that it may afterward go back to a previously experienced node (in (**c**), the green trajectory experienced after the blue one led to the addition of a new branch, with another reward node, while these two reward nodes corresponded to the same place in the maze).

	Mann-Whitney U-tests							
	Exploration vs Exploration Swimming Control				Exploitation vs Exploitation Swimming Control			
**Region**	*U*	*z*	*p*	*q*	*U*	*z*	*p*	*q*
Au1	113	0	1.0	1.03	107.5	0.087	0.93	0.96
PrL	100.5	-0.48	0.63	0.8	39.5	-2.8	4.5×10^{-3}	0.019
IL	87.5	-1	0.31	0.55	50.5	-2.4	0.018	0.045
Cg1	83.5	-1.2	0.24	0.9	64.5	-1.7	0.081	0.16
Cg2	106	-0.25	0.8	0.94	25.5	-3.4	5.6×10^{-5}	6.4×10^{-3}
RSD	85	-1.1	0.26	0.89	45	-2.6	9.3×10^{-3}	0.032
RSG	102	-0.41	0.68	0.82	34	-3.1	2.1×10^{-3}	0.014
Par	109.5	-0.1	0.92	0.97	30	-3.3	1.1×10^{-3}	9.7×10^{-3}
PostPar	88.5	-0.97	0.33	0.53	23	-3.6	3.7×10^{-4}	6.3×10^{-3}
MEC	86.5	-1.1	0.29	0.62	77	-0.94	0.35	0.47
DMS	98.5	-0.56	0.58	0.82	18.5	-3.8	1.7×10^{-4}	5.9×10^{-3}
DLS	79.5	-1.3	0.18	1.5	72.5	-1.4	0.16	0.25
AcbC	71	-1.7	0.089	3.02	99	-0.24	0.810	0.95
AcbS	108.5	-0.150	0.88	1.5	74	-1.3	0.18	0.27
VTA	144	1.3	0.2	1.30	117.5	0.52	0.60	0.76
SNc	99.5	-0.52	0.60	0.82	111	0.24	0.81	0.92
dCA1	98	-0.58	0.56	0.83	48	-2.5	0.014	0.039
dCA3	85.5	-1.10	0.27	0.71	46.5	-2.5	0.011	0.035
vCA1	85	-1.10	0.26	0.81	67	-1.60	0.10	0.18
vCA3	78	-1.40	0.16	1.80	34	-3.1	2.1×10^{-3}	0.012
dCA2	147	1.40	0.16	2.7	70	-1.50	0.13	0.21
dDG	138.5	1.10	0.29	0.58	76.5	-1.20	0.22	0.31
vDG	100	-0.50	0.62	0.81	98	-0.28	0.78	0.94
Lob IV/V	87	-1	0.30	0.57	68	-1.60	0.11	0.19
Lob VI	86	-1.10	0.28	0.68	34.5	-3.10	2.2×10^{-3}	0.011
Simplex	85.5	-1.1	0.27	0.77	87.5	-0.74	0.46	0.60
Crus I	88.5	-0.98	0.33	0.56	58	-2	0.042	0.09
Lob VII	58	-1.10	0.29	0.65	18	0	0.033	0.074
Crus II	81	-1.30	0.20	1.10	107.5	0.087	0.93	0.99
Lob IX	83.5	-1.20	0.24	1	89.5	-0.049	0.96	0.96
Lob X	112.5	0	1	1	110.5	0.22	0.83	0.91
Dent N	92.5	-0.81	0.42	0.65	65	-1.70	0.085	0.16
Fast N	116.5	0.15	0.88	0.97	49	-2.4	0.015	0.04
IntP N	142	1.20	0.23	1.10	40	-2.8	4.9×10^{-3}	0.018

Supplementary Table 1: Comparisons of c-Fos positive cell densities normalized on their respective swimming controls. False discovery rate (FDR) corrected p values, *q*, inferior to 0.05 are highlighted in red.

Abbreviations: cortex: primary auditory (Au1), prelimbic (PrL), infralimbic (IL), cingulate 1 and 2 (Cg1, Cg2), dysgranular and granular retrosplenial (RSD, RSG), parietal and posterior parietal (Par, PostPar), medial entorhinal (MEC); striatum and dopaminergic nuclei (DA nuc): dorsomedial striatum (DMS), dorsolateral striatum (DLS), nucleus accumbens core (AcbC) and shell (AcbS), ventral tegmental area (VTA), substantia nigra pars compacta (SNc); hippocampus: dorsal CA1 (dCA1), dorsal CA3 (dCA3), ventral CA1 (vCA1), ventral CA3 (vCA3), dorsal CA2 (dCA2), dorsal and ventral dentate gyrus (dDG, vDG); cerebellum: lobules IV/V (Lob IV/V), VI (Lob VI), VII (Lob VII), IX (Lob IX), X (Lob X), dentate (Dent N), fastigial (Fast N) and interpositus nuclei (IntP N).

	Kruskal-Wallis on exploration, exploitation, swimming controls and cage control			Mann-Whitney U-tests											
				Exploration vs Cage Control				Exploitation vs Cage Control				Exploration vs Exploitation			
**Region**	*H*	*p*	*q*	*U*	*z*	*p*	*q*	*U*	*z*	*p*	*q*	*U*	*z*	*p*	*q*
Au1	$H_{4,82}$ = 50.9	2.2×10^{-10}	6.3×10^{-10}	356	5.07	4.02×10^{-7}	1.17×10^{-6}	315	3.88	1.03×10^{-4}	2.05×10^{-4}	188	3.09	2.0×10^{-3}	5.81×10^{-3}
PrL	$H_{4,82}$ = 63.8	4.6×10^{-13}	5.2×10^{-12}	360	5.18	2.2×10^{-7}	8.79×10^{-7}	359	5.15	2.56×10^{-7}	1.37×10^{-6}	177	2.65	7.93×10^{-3}	1.59×10^{-2}
IL	$H_{4,82}$ = 64.1	3.9×10^{-13}	6.7×10^{-12}	360	5.18	2.19×10^{-7}	3.51×10^{-6}	356	5.05	4.37×10^{-7}	2.0×10^{-6}	186	3.03	2.46×10^{-3}	6.55×10^{-3}
Cg1	$H_{4,82}$ = 58.1	7.1×10^{-12}	4.0×10^{-11}	360	5.18	2.19×10^{-7}	2.34×10^{-6}	360	5.18	2.19×10^{-7}	3.51×10^{-6}	150	1.53	0.125	0.19
Cg2	$H_{4,82}$ = 59.5	3.6×10^{-12}	3.1×10^{-11}	360	5.18	2.19×10^{-7}	1.75×10^{-6}	360	5.18	2.19×10^{-7}	2.34×10^{-6}	109	-0.124	0.901	0.93
RSD	$H_{4,82}$ = 37.1	1.7×10^{-7}	2.4×10^{-7}	330	4.32	1.59×10^{-5}	2.12×10^{-5}	344	4.72	2.36×10^{-6}	8.38×10^{-6}	101	-0.456	0.648	0.741
RSG	$H_{4,82}$ = 38.4	9.1×10^{-8}	1.5×10^{-7}	343	4.69	2.71×10^{-6}	5.11×10^{-6}	341	4.63	3.59×10^{-6}	9.58×10^{-6}	85.5	-1.1	0.272	0.378
Par	$H_{4,82}$ = 58.7	5.4×10^{-12}	3.7×10^{-11}	360	5.18	2.2×10^{-7}	1.0×10^{-6}	360	5.18	2.2×10^{-7}	1.76×10^{-6}	127	0.56	0.575	0.708
PostPar	$H_{4,81}$ = 57.8	8.4×10^{-12}	4.1×10^{-11}	357	5.1	3.46×10^{-7}	1.11×10^{-6}	360	5.18	2.18×10^{-7}	6.99×10^{-6}	116	0.124	0.901	0.901
MEC	$H_{4,78}$ = 49.2	5.4×10^{-10}	1.4×10^{-9}	327	5	5.85×10^{-7}	1.56×10^{-6}	271	4.35	1.34×10^{-5}	2.86×10^{-5}	136	1.75	0.08	0.128
DMS	$H_{4,82}$ = 66.8	1.1×10^{-13}	3.6×10^{-12}	360	5.18	2.19×10^{-7}	1.4×10^{-6}	348	4.84	1.32×10^{-6}	5.3×10^{-6}	212	4.11	4.01×10^{-5}	6.42×10^{-4}
DLS	$H_{4,82}$ = 28.8	2.1×10^{-5}	2.8×10^{-5}	324	4.15	3.38×10^{-5}	4.33×10^{-5}	219	1.11	0.266	0.283	179	2.74	6.17×10^{-3}	1.41×10^{-2}
AcbC	$H_{4,82}$ = 38.1	1.1×10^{-7}	1.6×10^{-7}	344	4.72	2.35×10^{-6}	5.01×10^{-6}	248	1.95	0.0513	0.0608	197	3.47	5.29×10^{-4}	4.23×10^{-3}
AcbS	$H_{4,82}$ = 56.4	1.6×10^{-11}	6.2×10^{-11}	359	5.15	2.56×10^{-7}	9.1×10^{-7}	304	3.57	3.63×10^{-4}	6.83×10^{-4}	214	4.17	3.05 x 10-5	9.77×10^{-4}
VTA	$H_{4,82}$ =45.4	3.3×10^{-9}	8.1×10^{-9}	341	4.63	3.58×10^{-6}	5.2×10^{-6}	281	2.89	3.88×10^{-3}	6.21×10^{-3}	189	3.15	1.61×10^{-3}	5.72×10^{-3}
SNc	$H_{4,82}$ =2.4	0.66	0.66												
dCA1	$H_{4,82}$ = 39.8	4.8×10^{-8}	9.7×10^{-8}	342	4.65	3.35×10^{-6}	5.1×10^{-6}	333	4.4	1.07×10^{-5}	2.44×10^{-5}	130	0.705	0.481	0.641
dCA3	$H_{4,82}$ = 39.9	4.5×10^{-8}	9.6×10^{-8}	344	4.72	2.35×10^{-6}	4.7×10^{-6}	340	4.59	4.42×10^{-6}	1.09×10^{-5}	127	0.56	0.575	0.736
vCA1	$H_{4,82}$ = 57.6	9.3×10^{-12}	3.9×10^{-11}	360	5.18	2.19×10^{-7}	1.17×10^{-6}	359	5.15	2.56×10^{-7}	1.64×10^{-6}	145	1.31	0.191	0.278
vCA3	$H_{4,82}$ = 38.2	1.0×10^{-7}	1.5×10^{-7}	342	4.66	3.12×10^{-6}	4.99×10^{-6}	343	4.69	2.71×10^{-6}	7.88×10^{-6}	116	0.124	0.901	0.961
dCA2	$H_{4,82}$ = 11.7	0.019	0.020	246	1.89	0.0582	0.062	243	1.79	0.0733	0.0838	120	0.29	0.771	0.851
dDG	$H_{4,82}$ = 15.9	0.0032	0.0037	117	-1.82	0.0689	0.0711	85	-2.73	6.36×10^{-3}	9.25×10^{-3}	126	0.519	0.604	0.715
vDG	$H_{4,82}$ = 25.1	4.7×10^{-5}	5.9×10^{-5}	201	0.592	0.554	0.554	81.5	-2.83	4.65×10^{-3}	7.09×10^{-3}	191	3.22	1.3×10^{-3}	5.19×10^{-3}
Lob IV/V	$H_{4,82}$ = 55.9	2.1×10^{-11}	6.5×10^{-11}	360	5.18	2.19×10^{-7}	7.0×10^{-6}	266	2.47	0.0135	0.0181	208	3.94	8.12×10^{-5}	8.66×10^{-4}
Lob VI	$H_{4,78}$ = 56.1	1.8×10^{-11}	6.6×10^{-11}	300	4.98	6.23×10^{-7}	1.53×10^{-6}	292	4.7	2.58×10^{-6}	8.27×10^{-6}	196	3.44	5.75×10^{-4}	3.68×10^{-3}
Simplex	$H_{4,82}$ = 38.6	8.4×10^{-8}	1.5×10^{-7}	343	4.69	2.71×10^{-6}	4.82×10^{-6}	254	2.12	0.0338	0.0416	189	3.15	1.62×10^{-3}	5.18×10^{-3}
Crus I	$H_{4,82}$ = 39.2	6.2×10^{-8}	1.2×10^{-7}	343	4.69	2.71×10^{-6}	4.57×10^{-6}	263	2.37	0.0179	0.0229	176	2.61	8.97×10^{-3}	0.0169
Lob VII	$H_{4,55}$ = 33.7	8.6×10^{-7}	1.2×10^{-6}	152	4	6.35×10^{-5}	7.82×10^{-5}	90	3.2	1.35×10^{-3}	2.41×10^{-3}	91	2.79	5.3×10^{-3}	0.013
Crus II	$H_{4,81}$ = 13.5	9.1×10^{-3}	0.010	266	2.78	5.46×10^{-3}	6.47×10^{-3}	166	-0.194	0.846	0.846	178	2.68	7.45×10^{-3}	0.0159
Lob IX	$H_{4,76}$ = 38.3	9.5×10^{-8}	1.5×10^{-7}	289	4.6	4.22×10^{-6}	5.87×10^{-6}	217	3.17	1.53×10^{-3}	2.58×10^{-3}	149	2.35	0.0188	0.0317
Lob X	$H_{4,81}$ = 40.6	3.2×10^{-8}	7.2×10^{-8}	332	4.75	2.05×10^{-6}	4.68×10^{-6}	259	2.57	0.0102	0.0142	174	2.53	0.0114	0.0202
Dent N	$H_{4,80}$ = 3.9	0.42	0.42												
Fast N	$H_{4,80}$ =15.9	3.1×10^{-3}	3.8×10^{-3}	84.5	-2.48	0.0133	0.0152	214	1.5	0.134	0.147	33	-3.28	1.04×10^{-3}	5.55×10^{-3}
IntP N	$H_{4,80}$ =15.5	3.7×10^{-3}	4.2×10^{-3}	94	-2.18	0.0291	0.0322	183	0.542	0.588	0.607	33.5	-3.26	1.13×10^{-3}	5.16×10^{-3}

Supplementary Table 2: Comparisons of c-Fos positive cell densities normalized on cage controls. False discovery rate (FDR) corrected p values, *q*, inferior to 0.05 are highlighted in red.

Abbreviations: cortex: primary auditory (Au1), prelimbic (PrL), infralimbic (IL), cingulate 1 and 2 (Cg1, Cg2), dysgranular and granular retrosplenial (RSD, RSG), parietal and posterior parietal (Par, PostPar), medial entorhinal (MEC); striatum and dopaminergic nuclei (DA nuc): dorsomedial striatum (DMS), dorsolateral striatum (DLS), nucleus accumbens core (AcbC) and shell (AcbS), ventral tegmental area (VTA), substantia nigra pars compacta (SNc); hippocampus: dorsal CA1 (dCA1), dorsal CA3 (dCA3), ventral CA1 (vCA1), ventral CA3 (vCA3), dorsal CA2 (dCA2), dorsal and ventral dentate gyrus (dDG, vDG); cerebellum: lobules IV/V (Lob IV/V), VI (Lob VI), VII (Lob VII), IX (Lob IX), X (Lob X), dentate (Dent N), fastigial (Fast N) and interpositus nuclei (IntP N).

	Kruskal-Wallis on exploration, exploitation, swimming controls and cage control			Mann-Whitney U-tests											
				Exploration swimming control vs Cage Control				Exploitation swimming control vs Cage Control				Exploration swimming control vs Exploitation swimming control			
**Region**	*H*	*p*	*q*	*U*	*z*	*p*	*q*	*U*	*z*	*p*	*q*	*U*	*z*	*p*	*q*
Au1	$H_{4,82} = 50.9$	2.2×10^{-10}	6.3×10^{-10}	358	5.12	2.97×10^{-7}	1.19×10^{-6}	308.5	4.24	2.26×10^{-5}	9.03×10^{-5}	164.5	2.57	0.01	0.01
PrL	$H_{4,82} = 63.8$	4.6×10^{-13}	5.2×10^{-12}	360	5.18	2.19×10^{-7}	2.34×10^{-6}	333	4.98	6.42×10^{-7}	6.85×10^{-6}	189	3.64	2.68×10^{-4}	1.22×10^{-3}
IL	$H_{4,82} = 64.1$	3.9×10^{-13}	6.7×10^{-12}	360	5.18	2.19×10^{-7}	1.76×10^{-6}	330	4.88	1.02×10^{-6}	6.54×10^{-6}	193	3.82	1.33×10^{-4}	8.56×10^{-4}
Cg1	$H_{4,82} = 58.1$	7.1×10^{-12}	4.0×10^{-11}	360	5.18	2.19×10^{-7}	7.01×10^{-6}	335	5.04	4.68×10^{-7}	7.48×10^{-6}	178	3.16	1.55×10^{-3}	3.32×10^{-3}
Cg2	$H_{4,82} = 59.5$	3.6×10^{-12}	3.1×10^{-11}	360	5.18	2.19×10^{-7}	3.51×10^{-6}	336	5.07	3.99×10^{-7}	1.27×10^{-5}	179	3.21	1.34×10^{-3}	3.29×10^{-3}
RSD	$H_{4,82} = 37.1$	1.7×10^{-7}	2.4×10^{-7}	309.5	3.72	1.96×10^{-4}	2.51×10^{-4}	293.5	3.78	1.55×10^{-4}	4.95×10^{-4}	135	1.29	0.20	0.22
RSG	$H_{4,82} = 38.4$	9.1×10^{-8}	1.5×10^{-7}	311	3.77	1.65×10^{-4}	2.19×10^{-4}	262	2.83	4.66×10^{-3}	7.1×10^{-3}	146.5	1.79	0.073	0.09
Par	$H_{4,82} = 58.7$	5.4×10^{-12}	3.7×10^{-11}	357	5.09	3.48×10^{-7}	1.24×10^{-6}	325.5	4.75	2.02×10^{-6}	9.23×10^{-6}	180	3.25	1.15×10^{-3}	3.34×10^{-3}
PostPar	$H_{4,81} = 57.8$	8.4×10^{-12}	4.1×10^{-11}	331	4.92	8.71×10^{-7}	2.32×10^{-6}	329	4.86	1.18×10^{-6}	6.31×10^{-6}	169	3.24	1.2×10^{-3}	3.19×10^{-3}
MEC	$H_{4,78} = 49.2$	5.4×10^{-10}	1.4×10^{-9}	324	4.90	9.40×10^{-7}	2.31×10^{-6}	283	4.17	3.04×10^{-5}	1.08×10^{-4}	177	3.12	1.80×10^{-3}	3.62×10^{-3}
DMS	$H_{4,82} = 66.8$	1.1×10^{-13}	3.6×10^{-12}	359	5.15	2.56×10^{-7}	1.36×10^{-6}	267	2.98	2.87×10^{-3}	4.59×10^{-3}	210	4.56	5.08×10^{-6}	1.63×10^{-4}
DLS	$H_{4,82} = 28.8$	2.1×10^{-5}	2.8×10^{-5}	290	3.16	1.55×10^{-3}	1.91×10^{-3}	135.5	-0.97	0.33	0.35	168.5	2.75	5.94×10^{-3}	9.49×10^{-3}
AcbC	$H_{4,82} = 38.1$	1.1×10^{-7}	1.6×10^{-7}	326	4.20	2.66×10^{-5}	4.26×10^{-5}	245.5	2.33	0.02	0.027	169.5	2.79	5.21×10^{-3}	8.78×10^{-3}
AcbS	$H_{4,82} = 56.4$	1.6×10^{-11}	6.2×10^{-11}	355	5.04	4.71×10^{-7}	1.51×10^{-6}	240.5	2.18	0.029	0.037	200	4.12	3.72×10^{-5}	3.97×10^{-4}
VTA	$H_{4,82} = 45.4$	3.3×10^{-9}	8.1×10^{-9}	349.5	4.88	1.06×10^{-6}	2.43×10^{-6}	277.5	3.30	9.69×10^{-4}	2.07×10^{-3}	181.5	3.32	9.09×10^{-4}	3.23×10^{-3}
SNc	$H_{4,82} = 2.4$	0.66	0.66												
dCA1	$H_{4,82} = 39.8$	4.8×10^{-8}	9.7×10^{-8}	320	4.03	5.63×10^{-5}	8.58×10^{-5}	270	3.07	2.12×10^{-3}	3.58×10^{-3}	154.5	2.14	0.03	0.041
dCA3	$H_{4,82} = 39.9$	4.5×10^{-8}	9.6×10^{-8}	320	4.03	5.64×10^{-5}	8.20×10^{-5}	283	3.46	5.3×10^{-4}	1.3×10^{-3}	144.5	1.70	0.089	0.101
vCA1	$H_{4,82} = 57.6$	9.3×10^{-12}	3.9×10^{-11}	359	5.15	2.56×10^{-7}	1.17×10^{-6}	331	4.92	8.75×10^{-7}	7.00×10^{-6}	172	2.90	0.0037	6.58×10^{-3}
vCA3	$H_{4,82} = 38.2$	1.0×10^{-7}	1.5×10^{-7}	338	4.55	5.43×10^{-6}	9.66×10^{-6}	281	3.41	6.62×10^{-4}	1.51×10^{-3}	121	0.68	0.49	0.49
dCA2	$H_{4,82} = 11.7$	0.019	0.020	280	2.87	4.03×10^{-3}	4.78×10^{-3}	192	0.71	0.48	0.49	158	2.29	0.022	0.029
dDG	$H_{4,82} = 15.9$	0.0032	0.0037	131.5	-1.39	0.17	0.17	47.5	-3.63	2.79×10^{-4}	8.14×10^{-4}	159	2.33	0.019	0.027
vDG	$H_{4,82} = 25.1$	4.7×10^{-5}	5.9×10^{-5}	190	0.27	0.78	0.78	62.5	-3.18	1.48×10^{-3}	2.79×10^{-3}	185.5	3.49	4.79×10^{-4}	1.91×10^{-3}
Lob IV/V	$H_{4,82} = 55.9$	2.1×10^{-11}	6.5×10^{-11}	359	5.15	2.55×10^{-7}	1.63×10^{-6}	248.5	2.42	0.015	0.022	207	4.43	9.43×10^{-6}	1.51×10^{-4}
Lob VI	$H_{4,78} = 56.1$	1.8×10^{-11}	6.6×10^{-11}	300	4.98	6.23×10^{-7}	1.82×10^{-6}	231	3.17	1.54×10^{-3}	2.73×10^{-3}	199	4.08	4.49×10^{-5}	3.59×10^{-4}
Simplex	$H_{4,82} = 38.6$	8.4×10^{-8}	1.5×10^{-7}	338.5	4.56	5.073×10^{-6}	9.55×10^{-6}	245	2.31	0.02	0.027	174	2.99	2.79×10^{-3}	5.26×10^{-3}
Crus I	$H_{4,82} = 39.2$	6.2×10^{-8}	1.2×10^{-7}	337	4.52	6.24×10^{-6}	1.05×10^{-5}	237.5	2.09	0.037	0.043	192	3.77	1.59×10^{-4}	8.53×10^{-4}
Lob VII	$H_{4,55} = 33.7$	8.6×10^{-7}	1.2×10^{-6}	141	3.96	7.6×10^{-5}	1.06×10^{-4}	101.5	2.16	0.031	0.038	120	3.29	9.92×10^{-4}	3.17×10^{-3}
Crus II	$H_{4,81} = 13.5$	9.1×10^{-3}	0.010	244.5	2.14	0.033	0.037	171	0.29	0.77	0.77	145	1.72	0.085	0.10
Lob IX	$H_{4,76} = 38.3$	9.5×10^{-8}	1.5×10^{-7}	288.5	4.6	4.22×10^{-6}	8.42×10^{-6}	240.5	3.5	4.65×10^{-4}	1.24×10^{-3}	165	2.60	9.38×10^{-3}	0.014
Lob X	$H_{4,81} = 40.6$	3.2×10^{-8}	7.2×10^{-8}	334	4.80	1.52×10^{-6}	3.25×10^{-6}	264.5	3.23	1.25×10^{-3}	2.51×10^{-3}	178.5	3.19	1.44×10^{-3}	3.29×10^{-3}
Dent N	$H_{4,80} = 3.9$	0.42	0.42												
Fast N	$H_{4,80} = 15.9$	3.1×10^{-3}	3.8×10^{-3}	96	-2.12	0.034	0.037	116.5	-1.2	0.23	0.25	88	-0.72	0.47	0.49
IntP N	$H_{4,80} = 15.5$	3.7×10^{-3}	4.2×10^{-3}	103.5	-1.89	0.059	0.063	102	-1.67	0.094	0.108	132.5	1.18	0.24	0.25

Supplementary Table 3: Comparisons of c-Fos positive cell densities of swimming controls normalized on cage controls. False discovery rate (FDR) corrected p values, *q*, inferior to 0.05 are highlighted in red.
Abbreviations: cortex: primary auditory (Au1), prelimbic (PrL), infralimbic (IL), cingulate 1 and 2 (Cg1, Cg2), dysgranular and granular retrosplenial (RSD, RSG), parietal and posterior parietal (Par, PostPar), medial entorhinal (MEC); striatum and dopaminergic nuclei (DA nuc): dorsomedial striatum (DMS), dorsolateral striatum (DLS), nucleus accumbens core (AcbC) and shell (AcbS), ventral tegmental area (VTA), substantia nigra pars compacta (SNc); hippocampus: dorsal CA1 (dCA1), dorsal CA3 (dCA3), ventral CA1 (vCA1), ventral CA3 (vCA3), dorsal CA2 (dCA2), dorsal and ventral dentate gyrus (dDG, vDG); cerebellum: lobules IV/V (Lob IV/V), VI (Lob VI), VII (Lob VII), IX (Lob IX), X (Lob X), dentate (Dent N), fastigial (Fast N) and interpositus nuclei (IntP N).

	Sign test					
	Exploration mice			Exploitation mice		
**Region**	***Z***	***p***	***q***	***Z***	***p***	***q***
Au1	Z_{15} = -2.58	0.0098	0.28	Z_{15} = 0.52	0.61	0.88
PrL	Z_{15} = -0.80	0.42	0.94	Z_{15} = 1.55	0.12	0.43
IL	Z_{15} = -1.03	0.30	1.09	Z_{15} = 1.55	0.12	0.43
Cg1	Z_{15} = 0.00	1.00	1.32	Z_{15} = 0.52	0.61	0.88
Cg2	Z_{15} = 0.52	0.61	1.11	Z_{15} = 2.07	0.039	0.38
RSD	Z_{15} = -1.03	0.30	0.97	Z_{15} = 0.52	0.61	0.88
RSG	Z_{15} = 0.00	1.00	1.26	Z_{15} = 0.52	0.61	0.88
Par	Z_{15} = 0.52	0.61	1.03	Z_{15} = 0.00	1.00	1.00
PostPar	Z_{15} = -0.52	0.61	0.98	Z_{14} = 0.80	0.42	0.88
MEC	Z_{14} = -0.80	0.42	0.88	Z_{12} = 1.44	0.15	0.43
DMS	Z_{15} = 0.80	0.42	0.82	Z_{15} = 2.07	0.039	0.38
DLS	Z_{15} = 0.00	1.00	1.21	Z_{15} = 0.00	1.00	1.00
AcbC	Z_{15} = -1.55	0.12	1.76	Z_{15} = 2.07	0.04	0.38
AcbS	Z_{15} = 1.55	0.12	1.17	Z_{15} = 0.00	1.00	1.00
VTA	Z_{15} = 1.55	0.12	0.88	Z_{15} = 0.52	0.61	0.88
SNc	Z_{14} = 1.34	0.18	0.88	Z_{13} = 0.00	1.00	1.00
dCA1	Z_{15} = 0.52	0.61	0.92	Z_{15} = 0.00	1.00	1.00
dCA3	Z_{15} = 0.27	0.79	1.09	Z_{15} = 1.03	0.30	0.67
vCA1	Z_{15} = 0.00	1.00	1.16	Z_{14} = 0.27	0.79	0.99
vCA3	Z_{15} = 1.55	0.12	0.70	Z_{15} = 1.03	0.30	0.67
dCA2	Z_{13} = 0.00	1.00	1.11	Z_{15} = 1.03	0.30	0.67
dDG	Z_{15} = 0.00	1.00	1.07	Z_{15} = 0.52	0.61	0.88
vDG	Z_{15} = -1.34	0.18	0.75	Z_{15} = 1.55	0.12	0.43
Lob IV/V	N/A	N/A	N/A	N/A	N/A	N/A
Lob VI	N/A	N/A	N/A	N/A	N/A	N/A
Simplex	Z_{15} = -1.04	0.30	0.87	Z_{15} = 1.55	0.12	0.43
Crus I	Z_{15} = -1.04	0.31	0.79	Z_{15} = 0.00	1.00	1.00
Lob VII	N/A	N/A	N/A	N/A	N/A	N/A
Crus II	Z_{15} = 0.52	0.61	0.88	Z_{12} = 1.44	0.16	0.43
Lob IX	N/A	N/A	N/A	N/A	N/A	N/A
Lob X	N/A	N/A	N/A	N/A	N/A	N/A
Dent N	Z_{9} = 0.00	1.00	1.04	Z_{12} = 1.44	0.15	0.99
Fast N	Z_{9} = -1.03	0.32	0.73	Z_{12} = 0.29	0.77	0.43
IntP N	Z_{9} = 0	1.00	1.00	Z_{12} = 0.29	0.77	0.99

Supplementary Table 4: c-Fos positive cell densities left-right comparisons. No differences were detected between left and right hemispheres in exploration and exploitation mice. *q* values show p values corrected for False Discovery Rate (FDR). For lobules IV/V, VI, VII, IX, X, which are located in the vermal part of the cerebellum, there was no separate counting for left and right.

Abbreviations: cortex: primary auditory (Au1), prelimbic (PrL), infralimbic (IL), cingulate 1 and 2 (Cg1, Cg2), dysgranular and granular retrosplenial (RSD, RSG), parietal and posterior parietal (Par, PostPar), medial entorhinal (MEC); striatum and dopaminergic nuclei (DA nuc): dorsomedial striatum (DMS), dorsolateral striatum (DLS), nucleus accumbens core (AcbC) and shell (AcbS), ventral tegmental area (VTA), substantia nigra pars compacta (SNc); hippocampus: dorsal CA1 (dCA1), dorsal CA3 (dCA3), ventral CA1 (vCA1), ventral CA3 (vCA3), dorsal CA2 (dCA2), dorsal and ventral dentate gyrus (dDG, vDG); cerebellum: lobules IV/V (Lob IV/V), VI (Lob VI), VII (Lob VII), IX (Lob IX), X (Lob X), dentate(Dent N), fastigial (Fast N) and interpositus nuclei (IntP N).

a

Mouse	Path Integration				Model-based Reinforcement Learning				Model-free Reinforcement Learning (without memory of past actions)			
	Learning rate (η)	Error accumulation (σ)	Exploration/ exploitation trade-off (β)	Max (log likelihood)	Learning rate (η)	Discount factor (γ)	Exploration/ exploitation trade-off (β)	Max (log likelihood)	Learning rate (η)	Discount factor (γ)	Exploration/ exploitation trade-off (β)	Max (log likelihood)
1	0.001	0.0008	1.14	**-376.09**	0.47	0.93	4.43	**-353.48**	0.87	1.00	0.76	-345.85
2	0.001	1.0000	6.47	**-490.20**	0.13	1.00	6.58	**-472.41**	0.85	1.00	1.05	-421.82
3	0.002	0.0003	0.98	**-408.16**	0.39	0.78	6.93	**-379.45**	0.16	1.00	5.92	**-340.73**
4	0.001	0.0015	1.28	**-348.19**	0.89	0.87	4.83	**-328.25**	0.20	1.00	6.05	**-302.24**
5	0.001	0.0003	0.83	**-444.17**	0.80	0.97	3.03	**-423.04**	0.17	0.84	4.92	**-379.34**
6	0.996	0.0009	0.70	**-538.02**	0.76	0.29	200.00	**-503.53**	0.08	1.00	9.41	**-482.90**
7	0.001	1.0000	7.83	**-514.61**	0.89	0.88	4.39	**-491.09**	0.15	1.00	3.68	-484.85
8	0.982	0.0031	1.29	**-611.51**	0.89	0.90	4.25	**-576.73**	0.36	1.00	1.50	-552.72
9	1.000	0.0019	1.29	**-518.58**	0.76	0.96	3.53	**-466.68**	0.31	1.00	2.86	-417.21
10	0.001	0.0003	0.89	**-563.74**	0.75	0.28	200.00	**-519.95**	0.08	1.00	6.55	**-502.52**
11	0.628	0.0013	0.93	**-646.86**	0.88	0.56	11.69	**-639.53**	0.19	1.00	2.56	**-589.24**
12	0.001	0.9568	5.38	**-812.72**	0.80	0.94	3.50	**-750.07**	0.12	1.00	3.24	-743.17
13	0.936	0.0024	1.47	**-599.09**	0.97	0.68	8.93	**-579.27**	0.19	1.00	2.13	-576.56
14	0.001	0.0017	0.93	**-495.97**	0.32	0.85	4.96	-469.88	0.11	1.00	4.89	**-473.38**
15	0.576	0.0041	1.34	**-756.02**	0.71	0.99	3.01	-691.71	1.00	1.00	0.20	**-745.84**

b

Mouse	Model-free Reinforcement Learning (without memory of past actions)			
	Learning rate (η)	Discount factor (γ)	Exploration/ exploitation trade-off (β)	Max (log likelihood)
1	0.45	1.00	0.34	**-332.66**
2	0.02	1.00	18.40	**-344.68**
3	0.07	1.00	2.04	**-355.87**
4	0.05	1.00	4.07	**-318.48**
5	0.03	1.00	3.15	**-426.17**
6	0.03	1.00	1.80	**-523.92**
7	0.64	1.00	0.47	**-395.15**
8	0.58	1.00	0.28	**-524.22**
9	0.68	1.00	0.31	**-407.27**
10	0.64	1.00	0.07	**-579.21**
11	0.83	1.00	0.12	**-604.87**
12	0.66	1.00	0.23	**-653.92**
13	0.97	1.00	0.15	**-534.31**
14	0.03	1.00	4.15	**-444.91**
15	0.90	1.00	0.14	**-671.09**

Supplementary Table 5: Parameter optimization results for the three models capable of learning the sequence (a) and for the model-free algorithm without memory (b). The parameters were optimized for each mouse and each model by identifying the set giving the maximum log likelihood. In red are highlighted the highest maximum log likelihood values across the four learning models and orange values highlight the highest values amongst the three model capable of learning the sequence (a). These optimized parameters were used for the learning simulations (Figure 3, Supplementary Figures 7) and the correlation analysis between the model-free reinforcement learning parameters and c-Fos densities (Figures 3, Supplementary Figure 8 and Supplementary Table 4).

a

	Learning rate (η) ρ	Learning rate (η) p	Discount factor (γ) ρ	Discount factor (γ) p	Exploration/exploitation trade-off (β) ρ	Exploration/exploitation trade-off (β) p
**Learning rate (η)**			0.077	0.802	-0.81	0.001
**Discount factor (γ)**	0.077	0.802			-0.15	0.615
**Exploration/exploitation trade-off (β)**	-0.81	0.001	-0.15	0.615		

(Column group header: Spearman correlation)

b

Region	Learning rate (η) ρ	p	q	Discount factor (γ) ρ	p	q	Exploration/exploitation trade-off (β) ρ	p	q
Au1	0.45	0.125	0.250	-0.15	0.615	0.804	-0.04	0.91	0.967
PrL	0.61	0.030	0.117	0.15	0.615	0.804	-0.34	0.263	0.419
IL	0.39	0.189	0.292	0.00	1	1.000	-0.09	0.778	0.853
Cg1	0.03	0.921	0.921	-0.23	0.447	0.804	0.26	0.394	0.515
Cg2	0.59	0.036	0.122	0.15	0.615	0.804	-0.41	0.169	0.391
RSD	0.48	0.097	0.220	-0.08	0.802	0.880	-0.42	0.157	0.391
RSG	0.68	0.014	0.079	-0.15	0.615	0.804	-0.55	0.055	0.208
Par	0.14	0.643	0.754	0.15	0.615	0.804	0.00	1	1.000
PostPar	-0.12	0.71	0.779	-0.31	0.305	0.804	0.37	0.216	0.419
MEC	0.08	0.80	0.824	0.48	0.114	0.804	-0.41	0.184	0.391
DMS	0.30	0.315	0.412	-0.15	0.615	0.804	-0.36	0.231	0.419
DLS	-0.15	0.63	0.754	0.08	0.802	0.880	-0.23	0.448	0.564
AcbC	0.54	0.058	0.155	0.15	0.615	0.804	-0.69	0.012	0.074
AcbS	0.34	0.263	0.363	0.00	1	1.000	-0.28	0.353	0.480
VTA	0.54	0.06	0.155	-0.31	0.305	0.804	-0.33	0.271	0.419
SNc	0.53	0.064	0.155	0.23	0.447	0.804	-0.68	0.013	0.074
dCA1	0.70	0.01	0.068	-0.15	0.615	0.804	-0.55	0.052	0.208
dCA3	0.62	0.027	0.117	0.31	0.305	0.804	-0.78	0.003	0.026
vCA1	0.42	0.16	0.264	-0.08	0.802	0.880	-0.44	0.135	0.391
vCA3	0.73	0.006	0.060	0.23	0.447	0.804	-0.77	0.003	0.026
dCA2	0.55	0.055	0.155	-0.23	0.447	0.804	-0.65	0.018	0.087
dDG	0.29	0.343	0.432	-0.15	0.615	0.804	-0.11	0.723	0.819
vDG	0.09	0.765	0.813	-0.31	0.305	0.804	0.28	0.353	0.480
Lob IV/V	0.73	0.007	0.060	-0.23	0.447	0.804	-0.46	0.115	0.391
Lob VI	0.42	0.152	0.264	0.08	0.802	0.880	-0.40	0.178	0.391
Simplex	0.46	0.115	0.244	-0.23	0.447	0.804	-0.34	0.255	0.419
Crus I	0.75	0.004	0.060	-0.23	0.447	0.804	-0.41	0.169	0.391
Lob VII	0.50	0.267	0.363	0.41	0.363	0.804	-0.54	0.236	0.419
Crus II	0.36	0.224	0.331	-0.23	0.447	0.804	-0.31	0.297	0.439
Lob IX	0.45	0.163	0.264	-0.20	0.555	0.804	-0.18	0.595	0.723
Lob X	0.43	0.140	0.264	-0.39	0.193	0.804	-0.14	0.643	0.754
Dent N	0.61	0.031	0.117	0.08	0.802	0.880	-0.80	0.002	0.026
Fast N	0.74	0.005	0.060	0.00	1	1.000	-0.84	0.0006	0.020
IntP N	0.12	0.69	0.779	-0.23	0.447	0.804	0.00	1	1.000

(Column group header: Spearman correlation)

Supplementary Table 6: Spearman correlation analysis on the model-free reinforcement learning's parameters. Only the 13 mice for which the model-free reinforcement learning with memory yielded a maximum log likelihood value were included (i.e. mice 14 and 15 were excluded). **(a)** Correlation between the parameters. *p* levels inferior or equal to 0.05 are highlighted in red. (**b**) Correlation between the learning parameters and c-Fos densities. False discovery rate (FDR) corrected p values, *q*, inferior to 0.05 are highlighted in red. *q* values inferior to 0.07 are highlighted in orange.

Abbreviations: cortex: primary auditory (Au1), prelimbic (PrL), infralimbic (IL), cingulate 1 and 2 (Cg1, Cg2), dysgranular and granular retrosplenial (RSD, RSG), parietal and posterior parietal (Par, PostPar), medial entorhinal (MEC); striatum and dopaminergic nuclei (DA nuc): dorsomedial striatum (DMS), dorsolateral striatum (DLS), nucleus accumbens core (AcbC) and shell (AcbS), ventral tegmental area (VTA), substantia nigra pars compacta (SNc); hippocampus: dorsal CA1 (dCA1), dorsal CA3 (dCA3), ventral CA1 (vCA1), ventral CA3 (vCA3), dorsal CA2 (dCA2), dorsal and ventral dentate gyrus (dDG, vDG); cerebellum: lobules IV/V (Lob IV/V), VI (Lob VI), VII (Lob VII), IX (Lob IX), X (Lob X), dentate(Dent N), fastigial (Fast N) and interpositus nuclei (IntP N).
